# Quality of Life and Mental Health Among Families Caring for Children with Medical Complexity: A Scoping Review

**DOI:** 10.3390/healthcare14091124

**Published:** 2026-04-22

**Authors:** Ana Suárez-Carrasco, Álvaro León-Campos, Maria José Peláez-Cantero, Silvia García-Mayor, Bibiana Pérez-Ardanaz

**Affiliations:** 1 Pediatric Intensive Care Unit, Hospital Regional Universitario de Málaga, 29011 Málaga, Spain; anasuacar1@gmail.com; 2 Facultad de Ciencias de la Salud, Universidad de Málaga, 29071 Málaga, Spain; pelaez_mariajose@hotmail.com; 3 Nursing Department, Facultad de Ciencias de la Salud, Universidad de Málaga, 29071 Málaga, Spain; sgmayor@uma.es; 4 Complex Chronic and Palliative Paediatric Care Unit, Hospital Regional Universitario de Málaga, 29011 Málaga, Spain; 5 Facultad de Ciencias de la Salud, Universidad de Granada, 18016 Granada, Spain; bibianap@ugr.es

**Keywords:** multiple chronic conditions, chronic disease, caregivers, parents, quality of life, mental health, child

## Abstract

**Background:** Families caring for children with medical complexity (CMC) face sustained psychosocial demands that may impair health-related quality of life (HRQoL) and mental health. A clear map of how these outcomes are assessed and which factors shape them is needed to guide family-centered care. **Methods:** We conducted a scoping review following the Joanna Briggs Institute guidelines, and reports were prepared according to the PRISMA guidelines. Searches were conducted in PubMed, CINAHL, and EMBASE (January 2011 to December 2023) to find studies reporting on health-related quality of life (HRQoL) and/or mental health outcomes (anxiety, depression, burden) of family members and/or caregivers of CMC, including operationalization based on complex chronic condition (CCC) classifications, technology dependency, or the Pediatric Medical Complexity Algorithm (PMCA). Two reviewers independently screened records and recorded data, and the findings were synthesized narratively and thematically. **Results:** Sixty-seven studies met the inclusion criteria and spanned cross-sectional, cohort, case–control, pre–post and qualitative designs across conditions such as epilepsy, congenital heart disease, cerebral palsy, technology dependence and cancer. Common measures were PedsQL™ Family Impact Module, SF-36/12, HADS, Beck inventories and Zarit burden scales. Across the included studies, caregivers, predominantly mothers, frequently reported poorer HRQoL and higher levels of anxiety, depressive symptoms, or burden than comparison groups when these were available. Six recurrent themes emerged: (1) gendered caregiving with disproportionate maternal burden; (2) socio-economic gradients and financing models shaping outcomes; (3) culture, religion and spirituality as coping resources; (4) family and social support buffering distress; (5) school participation and coordinated services potentially reducing burden; and (6) interdependence between caregiver and child outcomes. **Conclusions:** Heterogeneous CMC definitions, outcome measures, and study designs limited comparability across studies. The mapped evidence suggests that family HRQoL and mental health outcomes are shaped by interacting clinical, social, and contextual factors. These findings may inform more family-centered and equity-oriented approaches to care. Future research should harmonize CMC definitions, standardize outcome measures, and prospectively evaluate multicomponent interventions.

## 1. Introduction

Advances in pediatric healthcare have significantly reduced infant mortality over the last century, while concurrently increasing the number of children living with chronic diseases, associated disabilities and medical complexity [1,2].

Complex chronic conditions (CCCs) in pediatrics refer to persistent and severe health conditions that significantly affect the quality of life of children and require ongoing medical management. These conditions not only demand prolonged treatments, but often significantly impact both the child and their family environment, making healthcare and psychosocial support essential [3,4]. Children with medical complexity (CMC) are, according to Cohen, a population that, regardless of underlying diagnoses, share characteristic patterns of health-related needs (health, psychosocial and/or community): the presence of severe medical conditions or high frailty; functional limitations that in many cases require technological assistance and/or neurological deterioration; and frequent healthcare use (more hospitalizations and follow-up with multiple specialists) [3,4,5]. Two complementary approaches are common in the literature: the diagnostic classification of Feudtner et al. for CCCs, and the Pediatric Medical Complexity Algorithm (PMCA) by Simon et al., which stratifies children by medical complexity and defines a “complex chronic disease” (C-CD) category which defines and stratifies those who present: (1) two or more chronic pathological conditions, with the involvement of two or more organs or systems and a long expected duration (greater than twelve months); (2) a progressive, debilitating disease, with decreased life expectancy; (3) a chronic condition with dependence on technology for a period greater than six months; or (4) neoplasms or metastatic diseases that affect vital functions, excluding those in remission for more than five years [6].

Although the included studies operationalized medical complexity using different frameworks—the most common being the classification of complex chronic conditions (CCCs) or the complex chronic disease (C-CD) category within the Pediatric Medical Complexity Algorithm (PMCA)—for consistency and unification of terminology, the target population is referred to as CMC as the primary term, regardless of the operational definition used.

CMC represent approximately 0.5–1% of the pediatric population and account for a disproportionate share of healthcare use and costs, consuming between 40% and 57% of pediatric hospital resources [7,8], and require high-intensity, coordinated care, including end-of-life needs [6,9]. Despite substantial use of health and social care services, families bear the greatest burden as they experience economic, social, and mental health problems [3,10], and often face fragmented pathways and unmet needs, underscoring the importance of home-centered care models [11,12].

Caring for a child with CMC requires parents to adapt to role strain, to assume new responsibilities [13], and to modify their lifestyles [9]. This requires the mobilization of resources from the entire family, mutual understanding and actions that promote adaptation to the new situation [14]. Furthermore, chronic and complex diseases are usually associated with a loss of normality and independence on the part of families, which can cause greater suffering [14]. Care focused on the home and the physical and psychological burden of the disease is usually accompanied by difficulty accepting the disease, a lack of social support, and also fatigue and stress [15], all a consequence of the permanent state of alert and a greater responsibility in care [14]. These sustained demands, together with family characteristics, contextual factors and other stressors, often mean caregivers’ own needs are overlooked [9,16]. Therefore, complex pediatric chronic disease has a considerable influence on mental health, family functioning and the quality of life of caregivers [14,17]. The family can become overburdened, exhausted and even collapse due to the demands of care, its own characteristics and its context [16]. Caring for a child with CMC places sustained demands on families, including complex care management, navigation of services and coordination with schools and community resources [9], with potential role strain, financial pressure and mental health symptoms that may compromise the family’s capacity to provide quality care [14,18].

Against this backdrop, understanding how family health-related quality of life (HRQoL) and mental health outcomes are assessed and what shapes them has become a priority.

Given the growing impact of CMC on health systems and families, a scoping review is warranted to map how HRQoL and mental health outcomes in families have been assessed, to identify associated factors, and to highlight evidence gaps, thereby informing practical areas for intervention by healthcare professionals. Given the heterogeneity of definitions, outcomes, and study designs in this field, a scoping review was considered the most suitable methodology to map existing evidence rather than to appraise study quality.

## 2. Methods

This scoping review was conducted to synthesize the published literature that evaluates the quality of life and mental health of families with CMC as well as to identify gaps in evidence and guide future lines of research and clinical intervention. This review followed the framework proposed by Peters et al. and adhered to the methodological guidance from the Joanna Briggs Institute (JBI) [19,20], and was conducted in accordance with the PRISMA guidelines. Although this review was not registered prior to initiation, the protocol has now been registered retrospectively in OSF, and the methods described herein were conducted according to this predefined protocol.

### 2.1. Research Question

Primary research question: What outcomes have been reported for family and caregiver HRQoL and mental health in the context of CMC, how are these outcomes measured, and which factors have been reported as being associated with them across settings and populations?

Population (P): Fathers and mothers (or primary family caregivers) of children and adolescents aged 0–25 years with medical complexity.

Concept (C): Health-related quality of life (HRQoL), mental health outcomes (anxiety, depression), and career burden.

Context (C): Pediatric inpatient, primary, and community-care settings providing services for CMC and their families.

### 2.2. Eligibility Criteria

To be included in this review, studies had to meet the following criteria:

Population: Mothers and fathers of children aged 0–25 years diagnosed with chronic complex diseases. We included a wide age range owing, on the one hand, to the intercultural nature of this study and, on the other hand, because these children are often assessed in pediatrics beyond the age of majority due to their clinical characteristics. Eligible studies included populations that could be classified within established frameworks, such as complex chronic conditions (CCCs) or the complex chronic disease (C-CD) category of the Pediatric Medical Complexity Algorithm (PMCA). In studies with samples of children with specific pathologies with varying levels of severity, only those who met the recognized criteria of medical complexity were included, as described by Simon et al. [8].
Outcomes: Studies evaluating quality of life, anxiety, depression, and caregiver burden.
Settings: Studies conducted in pediatric hospitals, specialized units, and primary care centers dedicated to patients with chronic complex conditions and their families.

Studies were excluded if they did not assess health-related quality of life, anxiety, depression, or caregiver burden in parents or primary caregivers; if the population focused on caregivers of people with chronic illnesses without meeting established definitions of medical complexity; if the caregivers themselves had a chronic illness that affected the patients’ quality of life; if the study design was qualitative or a non-systematic review without extractable empirical data relevant to the outcomes of interest; or if the reported data were incomplete for the purposes of this review.

### 2.3. Search Strategy

The search covered 1 January 2011–31 December 2023 across PubMed (National Library of Medicine, Bethesda, MD, USA), CINAHL (EBSCOhost, Ipswich, MA, USA), and EMBASE (Elsevier, Amsterdam, The Netherlands). Grey literature was not systematically searched because the review focused on peer-reviewed empirical evidence with validated outcome measures. Searches were limited to studies published in English or Spanish to ensure interpretability by all reviewers. Full strategies database outputs are provided in Supplementary Material Part S1.

The search strategy was not modified retrospectively to ensure consistency with the predefined protocol. Its adequacy was ensured by aligning the search terms and databases with the objectives of the scoping review, prioritizing sensitivity to capture studies on HRQoL and mental health outcomes in families of children with medical complexity. The strategy was peer-reviewed and piloted by two reviewers to confirm that key studies known to the team were captured, supporting its comprehensiveness and relevance to the review question.

### 2.4. Types of Studies

This scoping review included a broad range of study designs in order to comprehensively map the available evidence. The following types of sources were included:

- Analytical observational studies (prospective and retrospective cohort studies, case–control studies, and cross-sectional studies).
- Descriptive observational studies (case series, individual case reports, and descriptive cross-sectional studies).
- Experimental and quasi-experimental designs, considering pre-intervention baseline results.
- Qualitative studies and systematic reviews meeting the inclusion criteria.

Given the exploratory nature of scoping reviews, the inclusion of heterogeneous sources of evidence was considered. To ensure transparency and methodological coherence, the role of each type of source in the synthesis was predefined and explicitly structured.

Quantitative primary studies constituted the core evidence base of the review and were primarily used to identify outcomes, measurements instruments, and patterns in HRQoL and mental health among caregivers. Studies that used validated HRQoL or mental health instruments were prioritized in the synthesis of the results.

Qualitative studies were included to provide complementary evidence regarding caregiver experiences, emotional burden, coping processes, and contextual determinants. These studies were used to support and contextualize the thematic interpretation of the findings rather than to generate quantitative conclusions.

Systematic reviews were used only to identify previously reported trends, conceptual frameworks, and gaps in the literature. Their findings were not treated as primary empirical data in the synthesis.

Opinion papers were not included as primary sources of evidence and were excluded unless they provided extractable information relevant to the study objectives.

All included sources were integrated through a narrative and thematic synthesis. Editorials, commentaries without extractable information, conference abstracts, and studies reporting only child outcomes without family or caregiver measures were excluded.

### 2.5. Study Selection

**Figure 1:**
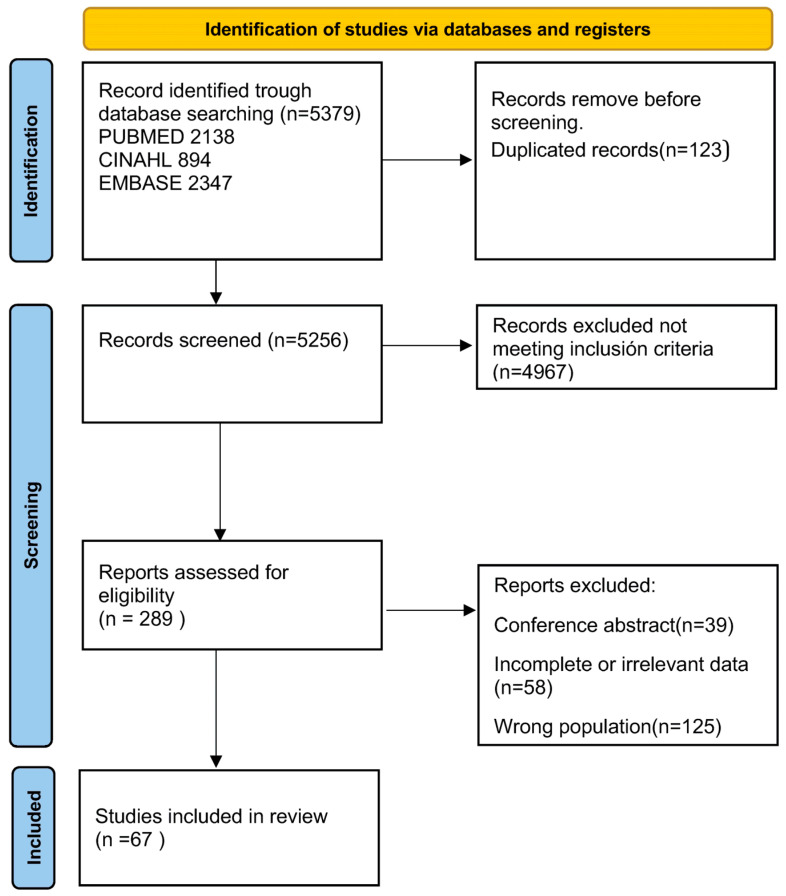
PRISMA-2020 flow diagram.

Each record was screened independently by two reviewers (ASC and ALC). Any discrepancies were resolved through discussion and, when required, adjudicated by a third reviewer (BPA). The PRISMA flow diagram in Figure 1 summarizes the selection process.

### 2.6. Data Extraction

**Figure 2:**
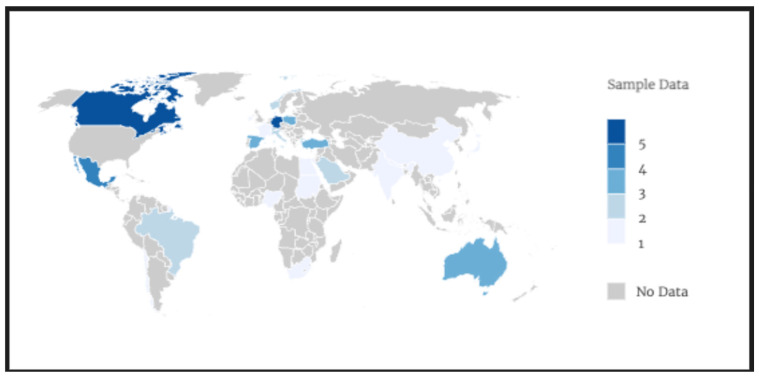
Geographical distribution. Note: Each color represents the range of studies from each region; n = 67.

Data extraction was performed independently by two reviewers using predefined charting tables. The data extracted included: publication year and authors; study design, objectives, and context; details of the children’s conditions and caregivers’ employment status; questionnaires and measures used; and results and key findings. We have attached a Supplementary File with the data extraction tables (Supplementary Material Part S2). Studies were categorized based on their main concepts and thematic areas. The extracted findings were initially open-coded by one reviewer to identify recurrent concepts related to caregiver mental health, quality of life, and contextual determinants. A second reviewer independently reviewed the codes to ensure consistency and conceptual coherence. Discrepancies in coding or categorization were resolved through discussion and consensus. Conceptually related codes were iteratively grouped into broader thematic categories, resulting in the final thematic areas used to organize the results. The analysis was conducted manually using structured data extraction tables; no qualitative analysis software was used. Additionally, the geographical distribution of the studies and the frequency and density of the thematic categories were analyzed (Figure 2). Studies originated mainly from North America (n = 28), Europe (n = 20), and Asia (n = 11), with limited evidence from Africa and Latin America (n = 8).

### 2.7. Quality Appraisal

Consistent with the methodological framework and exploratory objective of a scoping review, a formal risk-of-bias assessment was not conducted. The purpose of this review was not to evaluate the effectiveness of a specific intervention or to generate pooled estimates, but to systematically map the extent, characteristics, and measurement approaches of the available literature on HRQoL and mental health outcomes among families of children with medical complexity. However, to ensure the transparency and interpretability of the findings, the methodological characteristics of all included studies were carefully documented during the data-charting process. Information regarding study design, sample characteristics, outcome measures, and use of validated instruments was extracted for every study and used to contextualize the results.

### 2.8. Synthesis of Results

The findings were synthesized narratively and thematically, without quantitative pooling, consistent with the objectives of a scoping review.

## 3. Results

A total of 5379 studies were identified through database searches, including 2138 from PubMed, 894 from CINAHL, and 2347 from EMBASE. After removing duplicates, 5256 studies remained for screening. Following the initial screening based on inclusion criteria, 4972 studies were excluded. Subsequently, the eligibility of 289 full articles was assessed, of which 39 were excluded because they were conference abstracts, and 58 were excluded because they did not report outcomes relevant to the review objectives or provided incomplete or non-extractable data for family/caregiver HRQoL, anxiety, depression, or burden. This group included studies reporting only child outcomes, studies in which caregiver outcomes were not separately identifiable, and reports with insufficient data for extraction. Another 125 articles were excluded because they did not fit the study population or did not meet the complexity or age criteria. Finally, 67 studies met the inclusion criteria and were included (see Figure 1 for the PRISMA flow diagram).

### 3.1. Study Characteristics

The most frequent type of study included was cross-sectional (n = 43), followed by case–control (n = 9), cohort (n = 7), longitudinal (n = 3), and one pre–post intervention (n = 1). Two systematic reviews and two qualitative studies were also included.

Most of the available evidence derives from cross-sectional designs, which limits causal interpretation of the findings, whereas the smaller number of longitudinal and cohort studies provides more limited but relevant insights into changes over time.

**Figure 3:**
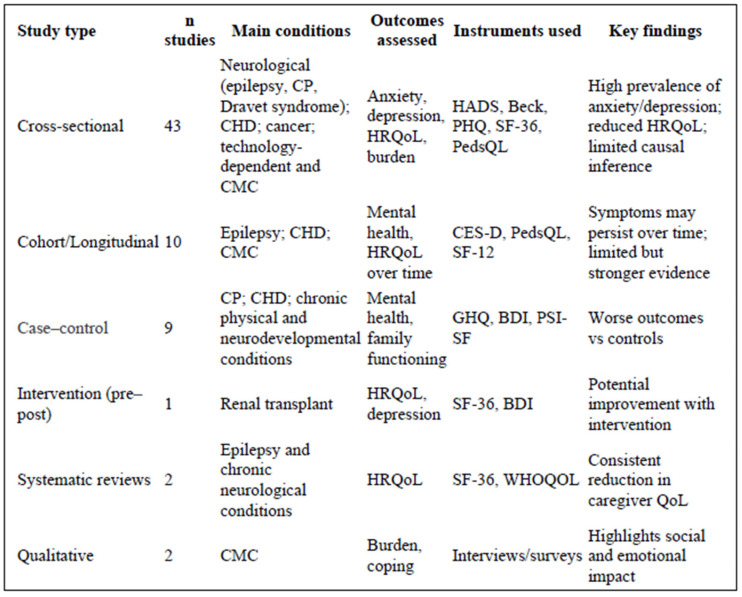
Summary of study designs, clinical conditions, outcomes assessed, measurement instruments, and key findings of included studies. Note: CP, cerebral palsy; CHD, congenital heart disease; CMC, children with medical complexity.

The conditions most frequently addressed were epilepsy, congenital heart diseases, cerebral palsy, technology dependence, and other chronic diseases, including cancer (see Figure 3).

### 3.2. Instruments Used

**Figure 4:**
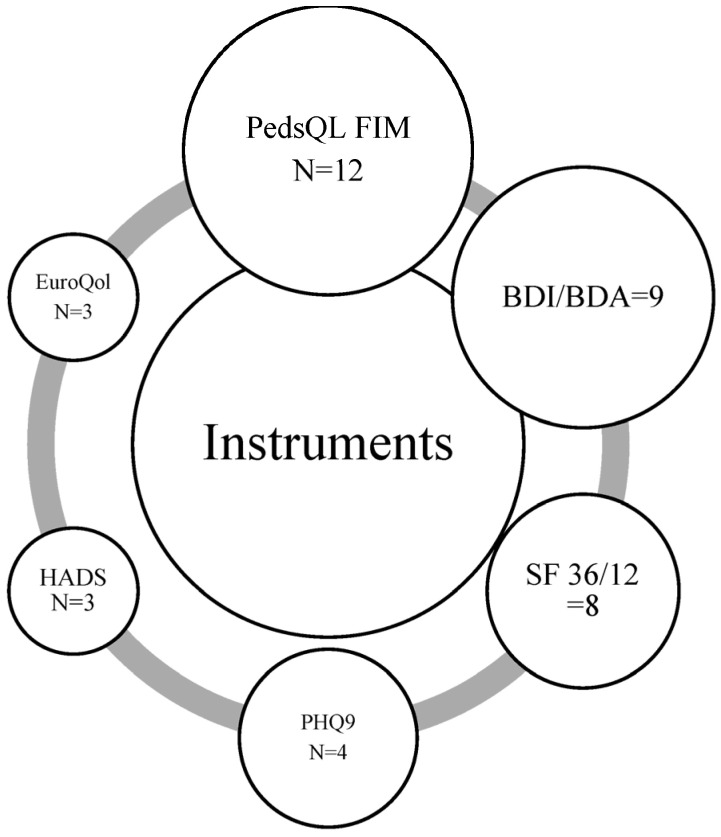
Instruments used to assess family/caregiver outcomes across included studies. Note: PedsQL FIM, Pediatric Quality of Life Inventory Family Impact Module; BDI/BAI, Beck Depression/Anxiety Inventory; SF-36/12, Short Form-36/12 Health Survey; PHQ9, Patient Health Questionnaire; HADS, Hospital Anxiety and Depression Scale; BDI/BAI, Beck Depression/Anxiety Inventory; EuroQol, EuroQol 5D 5L/EuroQol5D 3L/EuroQol VAS.

The most commonly used instruments to evaluate the outcomes were:

1. PedsQL™ Family Impact Module (FIM) (n = 12): Measures the impact of pediatric chronic illnesses on parents and family functioning, including physical, emotional, social, and cognitive functioning, as well as daily activities and communication [21].
2. Beck Depression and Anxiety Inventories (n = 10): Widely used self-report instruments designed to assess symptoms of depression and anxiety, respectively, with robust psychometric properties [22,23].
3. SF-36/12 (n = 8): Short Form Health Surveys (SF-36 and SF-12) evaluate overall health-related quality of life, covering physical and mental health domains, and are commonly used in both clinical and research settings [24,25].
4. Zarit Burden Interview (n = 6): Assesses caregiver burden, focusing on emotional, social, and financial stressors associated with caregiving responsibilities [26] (see Figure 4).

However, important heterogeneity in measurement tools was observed across studies, with some instruments focusing on overall family functioning (e.g., PedsQL™ FIM), while others assessed individual psychological symptoms (e.g., Beck inventories, HADS). This variability limits direct comparability of results across studies.

### 3.3. Mental Health and HRQoL Outcomes

Across the included studies, caregiver mental health outcomes were frequently assessed using validated instruments such as the HADS, Beck inventories, and PHQ-9. Anxiety and depression were common, particularly among mothers, with depressive symptoms reported for a substantial proportion of caregivers across studies; however, these findings were predominantly derived from studies with a cross-sectional design. Higher levels of anxiety and depression, as well as difficulties coping with the child’s illness, were frequently observed, and caregivers of children with complex medical conditions often reported intense and persistent psychological distress. Longitudinal evidence, although limited, suggests that these symptoms may persist over time rather than being transient [14,15,27,28,29,30].

Results from the PedsQL™ Family Impact Module (FIM) generally indicated lower HRQoL among caregivers of CMC compared with caregivers of children without chronic conditions These findings were consistent across studies using HRQoL-specific instruments, although the magnitude of impact varied depending on the tool and population studied.

However, a significant proportion experience a high or moderate burden, highlighting the variability of experiences. This variability appears to be influenced by both clinical and socio-demographic factors, as well as differences in measurement approaches.

Across conditions, patterns associated with poorer caregiver outcomes included high caregiving intensity, child functional limitations, and family socio-economic challenges. Overall, these findings suggest that different clinical conditions may impact caregivers through distinct mechanisms (e.g., emotional distress vs. care intensity), rather than through disease severity alone [31,32].

Selected examples further illustrate these patterns. In cystic fibrosis, depression symptoms were frequently reported among both mothers and fathers, suggesting notable mental health challenges in this group. Depressive symptoms were commonly reported among caregivers, often linked to fear of severe events such as sudden death. A substantial proportion of mothers reported major depression over follow-up periods, and high caregiver burden alongside anxiety and depression was reported.

These associations were more frequently reported in studies conducted in resource-limited settings or among populations with lower socio-economic status.

In addition to mental health challenges, studies consistently reported reduced quality of life among families of CMC compared with families of children without chronic conditions. Key findings included negative impacts on parents’ daily and social activities, especially among mothers, reduced emotional and social well-being within the family, and the need to give up personal or leisure activities in order to care for the child [29]. The results from the PedsQL™ Family Impact Module (FIM) generally indicated lower HRQoL among caregivers of CMC compared with caregivers of children without chronic conditions [9,16,18,27,33,34,35,36]. These findings were consistent across studies using HRQoL-specific instruments, although the magnitude of impact varied depending on the tool and population studied. In studies assessing caregiver burden using the Zarit Burden Interview, the results indicate that most caregivers experience mild or moderate levels of burden, while a significant proportion experience a high or moderate burden, highlighting the variability of experiences [16,18,33,34,35,36]. This variability appears to be influenced by both clinical and socio-demographic factors, as well as differences in measurement approaches.

Selected examples further illustrate these patterns. In cystic fibrosis, depression symptoms were frequently reported among both mothers and fathers, suggesting notable mental health challenges in this group [27]. In Dravet syndrome, depressive symptoms were commonly reported among caregivers, often linked to fear of severe events such as sudden death [37,38], while in epilepsy, a substantial proportion of mothers reported major depression over follow-up periods [39]. Among caregivers of children with cancer, high caregiver burden alongside anxiety and depression were reported [16]. Socio-cultural and economic factors also modified outcomes, with financial hardship and comorbidities associated with worse mental health and HRQoL scores in some settings [40,41]. Several studies further underscore the bidirectional relationship between caregiver mental health and child well-being, highlighting the importance of supporting both [27,42].

### 3.4. Key Findings

**Figure 5:**
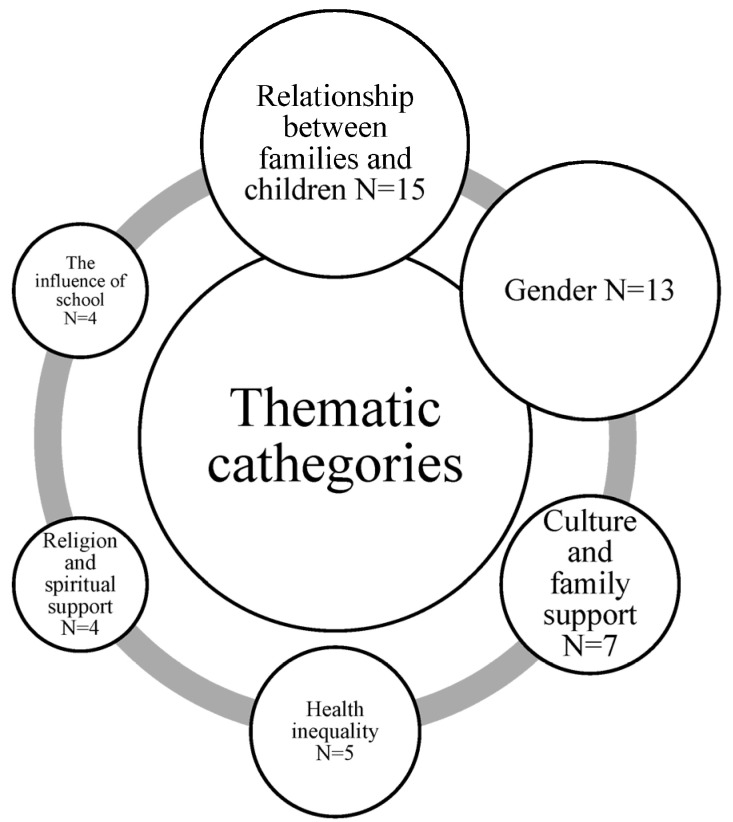
Tematic map of factors associated with family HRQoL and mental health (six themes). Note: Female caregiver proportion reported given the focus on gendered caregiving patterns in the included evidence.

This review identified six key thematic domains influencing caregiver HRQoL and mental health: gender roles in caregiving, socio-economic inequalities, spirituality and coping, family and social support, the role of school, and the interdependence between caregiver and child outcomes. These factors interact dynamically across the disease trajectory and highlight the need for early, family-centered interventions (Figure 5).

### 3.5. Mother Caregivers

It is essential to value the gender perspective in the care of CMC. In most articles, mothers represent a much higher percentage of care than fathers; therefore, they play a crucial role in this context. This pattern is particularly evident in cross-sectional studies, which constitute the majority of the available evidence. Women also show lower HRQoL [40] and worse mental health outcomes [30,43], with scores in some domains approaching those of CMC [29,44]. Differences were observed between high- and low-income countries: in the former, fathers appear slightly more involved in care. Employment status is relevant: many mothers leave or reduce employment, which affects socio-economic status and is a risk factor for unmet needs and for poorer HRQoL and mental health [45,46]. Overall, the predominance of cross-sectional designs limits causal interpretation, and longitudinal evidence on changes in caregiving roles over time remains limited.

### 3.6. Health Inequality

Socio-economic context plays an important role. Families with lower socio-economic status face greater financial difficulties and obtain lower HRQoL and mental health scores, affecting both families and CMC. This association is consistently observed across different study designs and measurement tools, although its magnitude varies by context. Across countries, differences are evident between universal, private and mixed health system models and resource-limited settings [40,41,45]. Studies conducted in resource-limited settings tend to report more pronounced disparities, suggesting a relevant role of structural factors. However, heterogeneity in the definition and measurement of socio-economic status (e.g., income, education, employment) limits direct comparability across studies. In addition, the predominance of cross-sectional evidence reduces the ability to determine the directionality of this relationship.

### 3.7. Religion and Spiritual Support

These findings are largely derived from qualitative and cross-sectional studies, and their interpretation is influenced by variability in how spirituality is conceptualized and measured. Overall, while spirituality appears to act as a protective factor, its mechanisms and consistency across populations remain insufficiently explored.

Several articles report beneficial associations of faith and spirituality with family and child HRQoL/mental health [29]. Faith is the set of religious beliefs; such beliefs may support more positive caregiving and self-management during stressful events [47]. Spirituality relates to meaning, purpose and connectedness, and may promote resilience, which in turn correlates with clinical/sociodemographic variables and with HRQoL and mental health [18,35].

This association is reported across different study types, although its magnitude varies depending on socio-cultural context. A higher educational level of caregivers has been associated with better HRQoL and more robust emotional/interpersonal ties; perceived family/social support relates to better psychological health, coping skills and lower burden [34,35,36,41,48]. However, differences in measurement approaches (e.g., perceived vs. structural support) introduce variability and limit comparability across studies.

### 3.8. The Influence of School

The impact of school is highly context-dependent, varying according to resource availability and institutional capacity. There is limited comparative evidence assessing different educational models or interventions.

School attendance is a major concern for families [41]. Education and socialization can improve children’s HRQoL [49,50] and can reduce care burden during school hours [50,51].

### 3.9. Relationship Between Family and Child Outcomes

Caregivers’ HRQoL and mental health are closely related to children’s outcomes. Multiple studies evaluate both family and child HRQoL/mental health in CMC [51,52,53]. Studies using paired caregiver/child measures tend to report stronger associations, particularly in mental health domains. Some prospective studies assess family mental health and HRQoL before diagnosis; findings vary, with some showing impairment [43] and others reporting inconclusive results [54]. Across the illness trajectory, impacts are evident, although domains and magnitude differ over time [54,55,56]. Longitudinal evidence, although limited, suggests that these relationships evolve over time and may differ across stages of the disease. These findings suggest that support strategies oriented to preserving family mental health and HRQoL may be most relevant when introduced early and adapted to the illness trajectory [57]. However, further longitudinal research is needed to clarify causal pathways and temporal dynamics.

## 4. Discussion

Although the included studies used different operational definitions to identify children with medical complexity (CMC), most relied on established frameworks such as complex chronic conditions (CCC) or the complex chronic disease category of the Pediatric Medical Complexity Algorithm (PMCA). This variability may influence the comparability of findings, particularly regarding caregiver burden and mental health outcomes.

For example, studies including children with high levels of technology dependence or intensive care needs may report greater caregiver strain than those identifying populations primarily based on diagnostic criteria. Therefore, the findings of this review should be interpreted cautiously, particularly when comparing results across studies or generalizing them to broader CMC populations.

Across studies, recurrent patterns emerged: mothers are frequently the primary caregivers and report greater challenges in terms of health-related quality of life and mental health; anxiety, depression, and perceived burden are common findings; and socio-economic status, family cohesion, and social support repeatedly modulate these outcomes. In addition, a recurring pattern of interdependence between caregiver well-being and child health was observed.

The findings also suggest that healthcare professionals may benefit from adopting multilevel and culturally sensitive approaches when analyzing the experiences of families caring for children with complex medical conditions. Nevertheless, these implications should be interpreted cautiously, as the review was not designed to evaluate the strength of evidence supporting specific determinants or interventions, which may help identify potential risk factors earlier and support timely interventions aimed at improving caregiver quality of life and mental health outcomes [4,36,58,59]. However, this interpretation is based on recurring themes across studies rather than on evidence of effectiveness. Similarly, the inclusion of “second burdens”, such as gender inequalities, socio-economic disadvantage, or limited social support, should be understood as frequently reported contextual factors rather than as confirmed predictors of caregiver outcomes [58]. This review highlights a recurrent gender imbalance in the caregiving role, with a higher proportion of women assuming this responsibility, often involving trade-offs in employment, leisure time, and self-care [14]. This pattern was described across several studies, although its magnitude and consistency varied depending on study design and context [36]. Socio-economic conditions were also frequently reported as influencing family well-being, particularly in relation to access to health services and social support. These findings should therefore be interpreted as commonly reported associations rather than as causal relationships.

Family self-efficacy and caregiver confidence were repeatedly described as factors related to better adaptation and lower perceived burden. However, given the methodological heterogeneity of the included studies, these findings should be interpreted as recurring themes in the literature rather than as conclusive evidence [57,60,61]. The same applies to the role of family advocacy, defined as active participation in medical, policy, and research decisions, which were often described as protective elements but were not consistently evaluated using comparable measures [13,60].

## 5. Limitations

This review has several limitations that should be considered:

Conceptual heterogeneity in the definition of CMC complicates study selection and substantially limits the comparability of the included studies, as differences in inclusion criteria can lead to variability both in the clinical complexity of the populations analyzed and in the outcomes, related to quality of life, mental health, and burden.

Heterogeneity in study designs, settings, and outcome measures also reduces the possibility of drawing consistent or comparable conclusions across studies, since different instruments and study populations may capture distinct aspects of caregiver well-being. Therefore, the findings of this review should be interpreted as an exploratory synthesis rather than as evidence of consistent relationships across studies.

This review was limited to studies published in English or Spanish, which may have introduced language bias.

Grey literature was not systematically searched, so relevant non-indexed or non-peer-reviewed evidence may not have been obtained.

The geographical distribution of the included studies was concentrated mainly in North America and Europe, which may limit the representation of other socio-economically and culturally diverse regions. This geographical imbalance may also influence the apparent consistency of some findings, as similar health systems and social contexts are overrepresented.

Taken together, these limitations indicate that the results should be interpreted with caution and primarily as a mapping of the existing literature rather than as definitive or comparable evidence. They also emphasize the need for greater conceptual clarity and methodological consistency in future research.

## 6. Conclusions

This review provides a structured synthesis of HRQoL and mental health outcomes among families of children with medical complexities and identifies recurrent themes and factors frequently described in the literature rather than consistent or causal relationships. These findings may contribute to informing family-centered care models, promoting home-based care, improving coordination, ensuring psychosocial support, and facilitating family training and empowerment, thus addressing families’ needs.

Key implications for practice include:

1. Ensuring universal, equitable access to healthcare.
2. Promoting professional competence for work in complex-care units.
3. Promoting family self-efficacy and advocacy.
4. Ensuring the inclusion of families in healthcare decisions.
5. Strengthening family and social support networks, facilitating access to resources aligned with to the psychological and mental health needs of both children and families.

However, these implications should be interpreted cautiously, particularly in light of the variability in study designs and measurement approaches.

Future research should prioritize more consistent definitions of CMC and facilitate an assessment of the impact of social determinants on caregivers’ mental health. Greater standardization of outcome measures and more specific studies that conduct a prospective evaluation of interventions designed to support caregivers’ well-being would also be necessary.

## Data Availability

No new data were created or analyzed in this study.

## Supplementary Materials

The following supporting information can be downloaded at: https://www.mdpi.com/article/10.3390/healthcare14091124/s1. The supplementary materials provide additional methodological details, including the complete search strategy for the scoping review (databases searched, search terms, and Boolean operators) and the comprehensive data extraction table presenting all variables and data extracted from each included study. Refs. [5,7,14,16,18,27,28,29,30,31,32,33,34,35,36,37,38,39,40,41,42,43,44,45,46,47,48,50,51,52,53,54,55,56,57,58,59,62,63,64,65,66,67,68,69,70,71,72,73,74,75,76,77,78,79,80,81,82,83,84,85,86,87,88,89,90,91,92,93] are cited in the supplementary material file.
