# Supplementary material for: Quality of Life and Mental Health Among Families Caring for Children with Medical Complexity: A Scoping Review
Source: Healthcare (Basel). 2026 Apr 22;14(9):1124. doi: 10.3390/healthcare14091124 (PMC13163436; doi:10.3390/healthcare14091124)
Supplement: Supplementary file 1 [file healthcare-14-01124-s001.zip › Supplementary material Part S1.pdf]

SEARCH METHODOLOGY

PUBMED

POPULATION

#1 "caregivers"[MeSH Terms] OR "caregivers"[All Fields] OR "carer"[All Fields] OR "carers"[All Fields] OR "carer's"[All Fields] ➔90,048

#2 "caregiver's"[All Fields] OR "caregivers"[MeSH Terms] OR "caregivers"[All Fields] OR "caregiver"[All Fields] OR "caregiving"[All Fields] ➔95,360

#3 "parent's"[All Fields] OR "parentally"[All Fields] OR "parentals"[All Fields] OR "parented"[All Fields] OR "parenting"[MeSH Terms] OR "parenting"[All Fields] OR "parents"[MeSH Terms] OR "parents"[All Fields] OR "parent"[All Fields] OR "parental"[All Fields] ➔457,402

#4 (#1 OR #2 OR #3) ➔543,718

EXPOSITION:

CHILDHOOD CHRONICITY

#5 (children with medical complexity OR disabled children) OR (polypathology[tiab] OR pluripathology[tiab] OR "complex chronic conditions" OR "complex medical conditions" OR ("Multimorbidity"[Mesh]) OR "Comorbidity"[Mesh] OR cooccurring illness*[tiab] OR "co-occurring illness*"[tiab] OR cooccurring morbid*[tiab] OR co-occurring morbid*[tiab] OR cooccurring patholog*[tiab] OR "co-occurring patholog*"[tiab] OR "multiple condition"[tiab] OR "multiple conditions"[tiab] OR "multiple diagnosis"[tiab] OR "multiple diagnoses"[tiab] OR "multiple disease"[tiab] OR "multiple diseases"[tiab] OR multiple health problem*[tiab] OR multiple illness*[tiab] OR "multiple pathology"[tiab] OR "multiple pathologies"[tiab] OR "Comorbidity"[Majr] OR "comorbidity" [tiab] OR "comorbidities"[tiab] OR "co-morbidity"[tiab] OR "co-morbidities"[tiab] OR multidisease[tiab] OR multi-disease[tiab] OR multi-diseases[tiab] OR multimorbidity[tiab] OR multimorbidities[tiab] OR multi-morbidity[tiab] OR multi-morbidities[tiab] OR multipathology[tiab] OR multipathologies[tiab] OR multi-pathology[tiab] OR multi-pathologies[tiab] OR pluripathology[tiab] OR pluripathologies[tiab] OR polypathology[tiab] OR polypathologies[tiab] OR poly-pathology[tiab] OR poly-pathologies[tiab] OR comorbid condition*[tiab] OR co-morbid condition*[tiab] OR comorbid diagnos*[tiab] OR co-morbid diagnos*[tiab] OR comorbid disease*[tiab] OR co-morbid disease*[tiab] OR "chronic disease"[MeSH Terms] OR ("chronic"[All Fields] AND "disease"[All Fields]) OR "chronic disease"[All Fields]) AND (child* OR pediatr* OR infant* OR adolesc*) ➔122,036

MULTIMORBIDITY

#6 polypathology[tiab] OR pluripathology[tiab] OR "complex chronic conditions" OR "complex medical conditions" OR ("Multimorbidity"[Mesh]) OR "Comorbidity"[Mesh] OR cooccurring illness*[tiab] OR "co-occurring illness*"[tiab] OR cooccurring morbid*[tiab] OR co-occurring morbid*[tiab] OR cooccurring

patholog*[tiab] OR “co-occurring patholog*”[tiab] OR “multiple condition”[tiab] OR “multiple conditions”[tiab] OR “multiple diagnosis”[tiab] OR “multiple diagnoses”[tiab] OR “multiple disease”[tiab] OR “multiple diseases”[tiab] OR multiple health problem*[tiab] OR multiple illness*[tiab] OR “multiple pathology”[tiab] OR “multiple pathologies”[tiab] OR “Comorbidity”[Majr] OR “comorbidity” [tiab] OR “comorbidities”[tiab] OR “co-morbidity”[tiab] OR “co-morbidities”[tiab] OR multidisease[tiab] OR multi-disease[tiab] OR multi-diseases[tiab] OR multimorbidity[tiab] OR multimorbidities[tiab] OR multi-morbidity[tiab] OR multi-morbidities[tiab] OR multipathology[tiab] OR multipathologies[tiab] OR multi-pathology[tiab] OR multi-pathologies[tiab] OR pluripathology[tiab] OR pluripathologies[tiab] OR polypathology[tiab] OR polypathologies[tiab] OR poly-pathology[tiab] OR poly-pathologies[tiab] OR comorbid condition*[tiab] OR co-morbid condition*[tiab] OR comorbid diagnos*[tiab] OR co-morbid diagnos*[tiab] OR comorbid disease*[tiab] OR co-morbid disease*[tiab] ➔ 278,105

OUTCOMES

#7 "quality of life"[MeSH Terms] OR ("quality"[All Fields] AND "life"[All Fields]) OR "quality of life"[All Fields] ➔459,726

#8 "physical"[tiab] OR "physically"[tiab] OR "physicals"[tiab] AND ("health"[MeSH Terms] OR "health"[tiab] OR "health s"[tiab] OR "healthful"[tiab] OR "healthfulness"[tiab] OR "healths"[tiab]) ➔218,893

#9 ("mental health"[MeSH Terms] OR ("mental"[All Fields] AND "health"[All Fields]) OR "mental health"[All Fields]) ➔431,714

#10 ("anxiety"[MeSH Terms] OR "anxiety"[All Fields] OR "anxieties"[All Fields] OR "anxiety s"[All Fields]) OR ("depressed"[All Fields] OR "depression"[MeSH Terms] OR "depression"[All Fields] OR "depressions"[All Fields] OR "depression s"[All Fields] OR "depressive disorder"[MeSH Terms] OR ("depressive"[All Fields] AND "disorder"[All Fields]) OR "depressive disorder"[All Fields] OR "depressivity"[All Fields] OR "depressive"[All Fields] OR "depressively"[All Fields] OR "depressiveness"[All Fields] OR "depressives"[All Fields]) ➔705,481

#11 "burden"[All Fields] OR "burdened"[All Fields] OR "burdening"[All Fields] OR "burdens"[All Fields] ➔287,656

#12 "sprains and strains"[MeSH Terms] OR ("sprains"[All Fields] AND "strains"[All Fields]) OR "sprains and strains"[All Fields] OR "strain"[All Fields] OR "strains"[All Fields] OR "strain's"[All Fields] ➔1,068,371

TYPE OF STUDIES

#13 "systematic review"[Publication Type] OR "systematic reviews as topic"[MeSH Terms] OR "systematic review"[All Fields] OR "meta-analysis"[Publication Type] OR "meta-analysis as topic"[MeSH Terms] OR "meta-analysis"[All Fields] ➔354,929

SEARCH STRING COMBINATION

#14 **QUALITY OF LIFE:** #4 AND #5 AND #6 AND #7 AND #8 ➔122

#15 **QUALITY OF LIFE REVIEWS:** #4 AND #5 AND #6 AND #7 AND #8 AND #13 ➔ 5

#16 **MENTAL HEALTH:** #4 AND #5 AND #6 AND #9 ➔ 1,458

#17 **MENTAL HEALTH REVIEWS:** #16 AND #13 ➔ 24

#18 **ANXIETY AND DEPRESSION** #4 AND #5 AND #6 AND #10➔2,045

#19 **ANXIETY AND DEPRESSION REVIEWS** #18 AND #13 ➔28

#20 **OVERLOAD** #4 AND #5 AND #6 AND #11 AND #12 ➔6

CINAHL

1.caregivers

2.carer

3.parents

4.1 OR 2 OR 3

5. ("children with medical complexity" OR "disabled children" OR polypathology OR pluripathology OR "complex chronic conditions" OR "complex medical conditions" OR "Multimorbidity") OR "Comorbidity" OR cooccurring illness* OR "co-occurring illness*" OR cooccurring morbid* OR co-occurring morbid* OR cooccurring patholog* OR "co-occurring patholog*" OR "multiple condition" OR "multiple conditions" OR "multiple diagnosis" OR "multiple diagnoses" OR "multiple disease" OR "multiple diseases"OR multiple health problem* OR multiple illness* OR "multiple pathology" OR "multiple pathologies" OR "Comorbidity" OR "comorbidity" OR "comorbidities" OR "co-morbidity" OR "co-morbidities" OR multidisease OR multi-disease OR multi-diseases OR multimorbidity OR multimorbidities OR multi-morbidity OR multi-morbidities OR multipathology OR multipathologies OR multi-pathology OR multi-pathologies OR pluripathology OR pluripathologies OR polypathology OR polypathologies OR poly-pathology OR poly-pathologies OR comorbid condition* OR co-morbid condition* OR comorbid diagnos* OR co-morbid diagnos* OR comorbid disease* OR co-morbid disease* OR chronic disease) AND (child* OR pediatr* OR infant* OR adolesc*)

6.Polypathology OR pluripathology OR "complex chronic conditions" OR "complex medical conditions" OR ("Multimorbidity) OR "Comorbidity OR cooccurring illness* OR "co-occurring illness*" OR cooccurring morbid* OR co-occurring morbid* OR cooccurring patholog* OR "co-occurring patholog*" OR "multiple condition"OR "multiple conditions" OR "multiple diagnosis" OR "multiple diagnoses" OR "multiple disease"OR "multiple diseases" OR multiple health problem* OR multiple illness* OR "multiple pathology" OR "multiple pathologies" OR "Comorbidity" OR "comorbidity"

OR "comorbidities" OR "co-morbidity" OR "co-morbidities" OR multidisease OR multi-disease OR multi-diseases OR multimorbidity OR multimorbidities OR multi-morbidity OR multi-morbidities OR multipathology OR multipathologies OR multi-pathology OR multi-pathologies OR pluripathology OR pluripathologies OR polypathology OR polypathologies OR poly-pathology OR poly-pathologies OR comorbid condition* OR co-morbid condition* OR comorbid diagnos* OR co-morbid diagnos* OR comorbid disease* OR co-morbid disease*

7. "quality of life"OR ("quality" AND "life") OR "quality of life"

8. "physical" OR "physically" OR "physicals" AND ("health" OR "health" OR "health s" OR "healthful" OR "healthfulness" OR "healths")

9. ("mental health" OR ("mental" AND "health") OR "mental health")

10. ("anxiety" OR "anxieties" OR "anxiety s") OR ("depressed" OR "depression" OR "depression" OR "depressions" OR "depression s" OR "depressive disorder"OR ("depressive"AND "disorder") OR "depressivity" OR "depressive" OR "depressively" OR "depressiveness" OR "depressives")

11. "burden"OR "burdened"OR "burdening"OR "burdens"

12. "sprains and strains" OR ("sprains" AND "strains") OR "strain" OR "strains" OR "strain's"

13. "systematic review"OR "systematic reviews as topic" OR "meta-analysis"OR "meta-analysis as topic"

<u>**SEARCH STRING COMBINATION**</u>

14. **QUALITY OF LIFE:** #4 AND #5 AND #6 AND #7 AND #8→127

15. **QUALITY OF LIFE REVIEWS:** #4 AND #5 AND #6 AND #7 AND #8 AND #13→9

16. **MENTAL HEALTH:** #4 AND #5 AND #6 AND #9→545

17. **MENTAL HEALTH REVIEWS:** #16 AND #13→6

18. **ANXIETY AND DEPRESSION** #4 AND #5 AND #6 AND #10→771

19. **ANXIETY AND DEPRESSION REVIEWS** #18 AND #13→18

20. **OVERLOAD** #4 AND #5 AND #6 AND #11 AND #12→44

EMBASE

#13'systematic review' OR 'systematic review as topic' OR 'meta-analysys' OR 'meta-analysis as topic'

#12'sprains and strains' OR ('sprains' AND 'strains') OR 'strain' OR 'strains' OR 'strain s'

#11'burden' OR 'burdened' OR 'burdening' OR 'burdens'

#10'anxiety' OR 'anxieties' OR 'anxietys' OR 'depressed' OR 'depression' OR 'depression s' OR 'depression s' OR 'depressive disorder' OR ('depressive' AND 'disorder') OR 'depressivity' OR 'depressive' OR 'depressively' OR 'depressiveness' OR 'depressives '

#9'mental' AND 'health' OR 'mental health'

#8('physical' OR 'physically' OR 'physicals') AND ('health' OR 'health s' OR 'healthful' OR 'healthfulness' OR 'healths')

#7'quality' AND 'life' OR 'quality of life'

#6 'complex chronic conditions' OR 'complex medical conditions' OR 'comorbidity or cooccurring illness*' OR 'co-occurring illness*' OR 'cooccurring morbid*' OR 'co-occurring morbid*' OR 'cooccurring patholog*' OR 'co-occurring patholog*' OR 'multiple condition' OR 'multiple conditions' OR 'multiple diagnosis' OR 'multiple diagnoses' OR 'multiple disease' OR 'multiple diseases' OR 'multiple health problem*' OR 'multiple illness*' OR 'multiple pathology' OR 'multiple pathologies' OR 'comorbidity' OR 'comorbidities' OR 'co-morbidity' OR 'co-morbidities' OR multidisease OR 'multi disease' OR 'multi diseases' OR multimorbidity OR multimorbidities OR 'multi morbidity' OR 'multi morbidities' OR multipathology OR multipathologies OR 'multi pathology' OR 'multi pathologies' OR pluripathology OR pluripathologies OR polypathology OR polypatholo gies OR 'poly pathology' OR 'poly pathologies' OR 'comorbid condition*' OR 'co-morbid condition*' OR 'comorbid diagnos*' OR 'co-morbid diagnos*' OR 'comorbid disease*' OR 'co-morbid disease*'

#5 ('children with medical complexity' OR 'disabled children' OR 'complex chronic conditions' OR 'complex medical conditions' OR 'multimorbidity' OR 'cooccurring illness*' OR 'co-occurring illness*' OR 'cooccurring morbid*' OR 'co occurring morbid*' OR 'cooccurring patholog*' OR 'co-occurring patholog*' OR 'multiple condition' OR 'multiple conditions' OR 'multiple diagnosis' OR 'multiple diagnoses' OR 'multiple disease' OR 'multiple diseases' OR 'multiple health problem*' OR 'multiple illness*' OR 'multiple pathology' OR 'multiple pathologies' OR 'comorbidity' OR 'comorbidities' OR 'co-morbidity' OR 'co-morbidities' OR multidisease OR 'multi disease' OR 'multi diseases' OR multimorbidity OR multimorbidities OR 'multi morbidity' OR 'multi-morbidities' OR multipathology OR multipathologies OR 'multi-pathology' OR 'multi-pathologies' OR pluripathology OR pluripathologies OR polypathology OR polypatholo gies OR 'poly-pathology' OR 'poly-pathologies' OR 'comorbid condition*' OR 'co-morbid condition*' OR 'comorbid diagnos*' OR 'co-morbid diagnos*' OR 'comorbid

disease*' OR 'co-morbid disease*' OR 'chronic disease') AND
(child* OR pediatr* OR infant* OR adolesc*)

#4#1 OR #2 OR #3

#3parents

#2carer

#1caregivers

SEARCH STRING COMBINATION

#14 **QUALITY OF LIFE:** #4 AND #5 AND #6 AND #7 AND #8→143

#15 **QUALITY OF LIFE REVIEWS:** #4 AND #5 AND #6 AND #7 AND #8 AND #13→4

#16 **MENTAL HEALTH:** #4 AND #5 AND #6 AND #9→949

#17 **MENTAL HEALTH REVIEWS:** #16 AND #13→27

#18 **ANXIETY AND DEPRESSION** #4 AND #5 AND #6 AND #10→1179

#19 **ANXIETY AND DEPRESSION REVIEWS** #18 AND #13→31

#20 **OVERLOAD** #4 AND #5 AND #6 AND #11 AND #12→14
