# Supplementary material for: Quality of Life and Mental Health Among Families Caring for Children with Medical Complexity: A Scoping Review
Source: Healthcare (Basel). 2026 Apr 22;14(9):1124. doi: 10.3390/healthcare14091124 (PMC13163436; doi:10.3390/healthcare14091124)
Supplement: Supplementary file 1 [file healthcare-14-01124-s001.zip › Supplementary materials Part S2.pdf]

Author, year	Place of study	Study design	Sample size	%Female	Employment	Pathology	Mean age	Questionnaires
Catherine Marx et al, 2011 [62]	Brazil	Cross-sectional study. Case-Control	N=45<br><br>N Control= 50	The prevalence of caregivers group was made up mainly of more single women..	Weekly work <20 hours Cases 78% Control 12%	Cerebral Palsy	6 month s-12 years old	Beck depression inventory(BDI) Escala de Epworth. Excessive daytime sleepiness(EDS) The state anxiety inventory STAI. STAI-T refers to relatively stable individual differences in the tendency to react to situations perceived as threatening, with increases in intensity or state of anxiety. The state anxiety inventory STAI-E in turn, analyzes subjective feelings of tension that can vary in intensity, according to the context
Kjersti Ramstad, 2012 [31]	Norway	Cross sectional (two groups)	n=105			Cerebral Palsy	14	General Health Questionnaire (GHQ-30)
Jennifer L. Hefner et al, 2013 [32]	US	Cross sectional study	N=122		Parental education High school degree or less 16. 13.0% Some college (, 4 yr) 43. 35.3% College graduate (> 4 yr) 59. 48.4%<br><br>Family finances It's a financial struggle 28 22.3% It's tight but we are doing fine 66. 54.1%. Finances aren't really a problem 26. 23.0%	Ventilator-dependent children	0–2r 26. 21.3%<br><br>3–15 59. 48.4%<br><br>16-30 37. 30.3 %	PHQ-2 = two-item Patient Health Questionnaire.<br><br>Caregiver burden daily

M. A. Landolt, 2014 [30]	Swiss	Longitudinal case–control	N=408<br><br>Normal population= 92 498	100%		Congenital heart disease		Hopkins Symptom Checklist (SCL-8)is designed to measure psychological distress with symptoms of anxiety and depression.
Maria Esposito et al, 2014 [63]	Italy	Cross-sectional, case-control cohort survey multicenter	N=37(cases)<br><br>N=405(controls)	100%		Neurofibromatosis 1	7,86 Cases<br><br>8.54 Controls	Parenting Stress Index-ShortForm (PSI-SF)
Alexandra L Quittner, 2014 [27]	US	Cross sectional study	Mothers N=3127<br><br>Fathers N=975	76,26%		Cystic Fibrosis		Hospital Anxiety and Depression Scale.(HADS 14-item)<br><br>Center for Epidemiologic Studies-Depression scale (CES-D)
Katarzyna Wojtas et al, 2014 [64]	Poland	Cross sectional study	N=213	69,5%		Epilepsy		HADS-M (Hospital Anxiety and Depression Scale Modified HADS-M, BSSS (Berlin Social Suport Scale), Inventory for Measurement Coping Mini-COPE and the author's questionnaire.
Xolyanetzin Montero Pardo et al,	Mexico	Cross sectional study	N=100	93%	Housewife 71%, 18% Employed 5%Merchants 5% Professional 4% Unemployed 2%	Cancer	8	Zarit Caregivers.<br><br>The anxiety and depression of Beck

2015 [16]								inventory.<br><br>The family support for the CPI scale.<br><br>The scale modes of coping with stress by Lazarus and Folkman
Mark A.Ferro et al, 2015 [65]	Canada	Data came from the National Longitudinal Survey of Children and Youth (NLSCY) Cases-Control	Chronic physical Illness N=1932<br><br>Healthy<br><br>N=8782	100%	Household Income ≥ $80,000% CPI→15,5<br><br>H→16,6<br><br>Post-secondary graduate, %<br><br>CPI→38,7<br><br>H→38,9	Chronic physical illness	10,5	Reduced version of the Center for Epidemiological Studies Depression Scale (CES-D)<br><br>12-item General Functioning subscale of the McMaster Family Assessment Device (FAD)
Rodrigo López et al, 2015 [58]	Chile	Prospective longitudinal. Cases-Control	Case n=40<br><br>Control n=115	Case=65%<br><br>Control=80,87 %	Poverty<br><br>Cases=50%<br><br>Control=39,13%	Congenital Heart Disease		General Health Questionnaire (GHQ-12),the Basic Psychological Needs Scales (BPN), the Self-Determination Scale (SDS),and the Beck Hopeless- ness Scale (BHS)
Sarah Johaningsmeir et al, 2015 [5]	US	Longitudinal study. Baseline, 1 year, 2	N=72			Children with medical complexity	3,8	Pediatric Quality of Life Inventory Family Impact Module (PedsQL FIM)<br><br>Pediatric Quality of Life Inventory

		year						Healthcare Satisfaction Module(PedsQL HCS)
Ahmad Azhar et al, 2016 [29]	Saudi Arabia	Cross Sectional study	Families 180	87,2%		Congenital Heart Diseases Simple 69,5% Complex 30,5%	5,65	Quality Of Life<br><br>BIS – biological impact score, PIS – psychological impact score, SIS – social impact score.
Bahar Büyükkarag öz et al, 2016 [66]	Turkey	Pre-post counselin g and education al program	Patients and Families (n=12)		Fathers in work 58,3%<br>Mothers housewives 91,3%	Renal Transplant	14.9	Beck Depression Inventory (BDI)<br><br>Rosenberg Self-Esteem Scale<br><br>Short-Form 36
Ozalp Ekinci et al, 2016 [28]	Turkey	Cross-sectional, case-control cohort survey	N=53	100%		Epilepsy	11.78	Beck Depression Inventory(BDI)<br><br>STAI state<br><br>STAI trait
Adeniran O. Okewole et al, 2016 [67]	Nigeria	Cross sectional study	N=100	90%	Employed 95%<br><br>Unemployed 5%	Neuropsychi atric disorders 63% seizure disorders 40%comorbi d diagnosis	11,6	Patient Health Questionnaire, PHQ-9
Soyong Eom et al, 2017 [54]	Korea	Cross sectional study	N=70			Mitochondri al Disease	4.60	

Krista Keilty et al, 2017 [68]	Canada	Prospective cohort study	Family caregivers of CMT (n=42) and caregivers of healthy children (n=43)	CMT 83,3%<br><br>Control 88,4%	Full time CMT35,7% Control 51,2%<br>Part time CMT 16,7% Control 27,9%<br>Unemployed CMT 38,1% Control 9,3%<br>Self-employed CMT 7,1% Control 9,3%<br>Retired CMT 2,5% Control 0%<br>On leave CMT 0% Control 2,3%	Children who depend on medical technology (CMT)	12 months-18 years	Octagonal Basic Motionlogger<br><br>Pittsburgh Sleep Quality Index (PSQI)<br><br>Short Form-12 Survey<br><br>Center for Epidemiological Studies Depression Scale (CES-D Scale)<br><br>Epworth Sleepiness Scale
Nicole Villas et al, 2017 [38]	US	Cross sectional study	N=256			Dravet syndrome	Median 7-10	Each survey included a demographic possible as well as content-related sections about characteristics, potencial comorbidities, medications and efficacy, and caregiver/family dynamics Responses included six-point Likert scales, lists, closed-question multiple choice, and open response.
Klajdi Puka et al, 2018 [39]	Canada	Sistematic Review	15 studies	(100%),<br>(76%) (72%)<br>(89%) (90%)<br>(88%) (85%)		Epilepsy	14,84	Short Form health survey (SF-36 or SF-12); evaluating health-related QOL), the World Health Organization Quality of Life Instrument-Abbreviated Version (WHOQOL-BREF), the European Health Interview Surveys (EUROHIS)-QOL-8, a single-item QOL measure , and the Parental

								QOL Difficulties Questionnaire
Klajdi Puka et al, 2019 [69]	Canada	Prospective cohort study	N=356	100%	At diagnosis 236 (67%)<br><br>10-Year fllow-up 123 (78%)	Epilepsy	At diagnosis 7,9<br><br>10-Year follow-up 18,1	The Center for Epidemiological Studies Depression Scale (CES - D)<br><br>The Family Inventory of Life Events and Changes (FILE)<br><br>Family APGAR<br><br>The Family Inventory of Resources for Management (FIRM)
Yuca Mori et al, 2018[55]	Australia	Longitudinal study. 2002,2006,2009, 2011	N=198	92,4%	Full-time homemaker 48,8%, 49,3%, 46,7%, 50, 3%Part-time employment 26,4%, 32,9%, 37,3%, 37,1% Full-time employment 24,8%, 17,9%, 16%, 12,6%	Rett Syndrome	<7y. 22,2% 7-11y. 25.8% 11-17y26.8% >17y. 25.3%	Short Form 12 Health Survey(SF-12)<br><br>McMaster Family Assessment Device (FAD)
Adam Strzelczyk et al, 2019[70]	Germany	Cross-sectional, prospective multicenter survey.	Caregivers (n=93)		Mothers in work 56%<br><br>Fathers in work 82%	Dravet syndrome	10.1	The Beck Depression Inventory (BDI) II depression scale<br><br>EuroQol-5D-3L(EQ-5D-3L)<br><br>EuroQol-Visual Analog Scale(EQ-VAS)<br><br>Total cost

Ellison Suthoff et al, 2019 [71]	Germany, Ireland , United Kingdom, and the US	Prospective observational international cohort study.	Caregivers (n= 88)	(85%	Employed full time 34%(n=29)<br>Employed part time 21%(n=18)<br>Unable to work for CF 10%(n=9)<br>Unemployed 35%(n=30)	Cystic Fibrosis	11,9	Short Form Survey-12(SF-12)<br><br>The Work Productivity and Activity Impairment: Specific Health Problem V2.0 (WPAI:SHP)<br><br>The Caregiver Quality of Life Cystic Fibrosis (CQOLCF)<br><br>The Multidimensional Scale of Perceived Social Support (MSPSS)<br><br>The Child Health Questionnaire–Parent Form 28 (CHQ- PF28)
Mayra L. Almanza-Sepulveda, 2019 [45]	Canada	Cross sectional study	N=86	81,40%	Employed 66,30%<br><br>Not employed 33,70%	Epilepsy	11,87	Caregiver depressive symptoms QIDS, Caregiver anxiety GAD-7. Family Adaptability, Partnership, Growth, Affection, and Resolve (Family APGAR), Family Inventory of Resources for Management (FIRM).. Family demands Family Inventory of Life Events and Changes (FILE)
Margot A. Lazow, 2019 [72]	US	Cross sectional study	N=33	67%	Employed 75%<br><br>Stay at home parent and/or retired 16%<br><br>Unemployed and or disable 9%	Osteogenesis imperfecta	9,2	Pediatric Inventory for Parents (PIP) PedsQL Family Impact Module (FIM) Depressive symptoms were assessed using the Center for Epidemiologic Studies Depression Scale (CES-D)

Fadwa M. S. Mohammed et al ,2019 [47]	Sudan	Cross-sectional facility - based study	N=65	92,3%	Housewife 89,2%<br><br>Teacher 6,2%<br><br>Other 4,6%	Cerebral Palsy	4-7y 64,6%<br><br>8-11y 21,5%<br><br>12-15y13, 8%	QoL of caregivers 4 domains: Physical, social, support for caring, and nancial burden.
Dominique L. Denniss et al, 2019 [52]	Australia	Cross sectional study	N=87	100%	Gross weekly household income Below national average 60% Above national average 31%	Complex Congenital Heart Disease. Single ventricle group(n=30) Biventricular group (n=57)	2,7	Pediatric Quality of Life Inventory, which assessed the outcomes of child and maternal HRQOL. (PedsQL FIM) Maternal HRQOL and family functioning summary scores.(36 items)<br><br>Depression Anxiety Stress Scales DASS (21 items),
Akanksha Rani et al, 2019 [41]	India	Cross-sectional descriptive study	N=60	Mothers N= 17 28,3%<br><br>Fathers N=43, 71,7%<br><br>Mothers principal caregiver 85%.	Unemployed/homemaker 18,3% Unskilled 13,3% Semi-skilled 43,3% Skilled 25%	Epilepsy	8,4	Parenting Stress Inventory(PSI)<br><br>Parent Stigma Scale

Janna Riechmann et al,2019 [46]	Germany	Cross-sectional study	N=489		The overall employment rate of mothers (60.5%) and fathers (81.2%) was high	Epilepsy	10,4	EuroQol 5D VAS<br><br>Beck Depression In- ventory II (BDI-II)
Filiberto Toledano-Toledano et al, 2019 [36]	Mexico	Cross sectional study	N=416	81,7%	Unpaid work (exclusively devoted to home or studies) Woman 80,88% Men 2,63%<br><br>Paid work Woman 15,88% Men 75%<br><br>Unemployed Woman 3,24% Men 22,37%	Chronic diseases	5,91	Family Support Questionnaire, Social Support Network Scale, Zarit Burden Interview, Parental Stress Scale, Family Functioning Scale, World Health Organization Well-Being Index, Historic-Psycho-Socio-Cultural-Premises Scale (HSCPs)
, Oscar Werner et al, 2019 [42]	France	Cross - sectional study	Fathers= 49<br><br>Mothers =71<br><br>Children =73	59,16%		Congenital Heart Diseases	11 months	The parental level of anxiety was assessed using a visual analog scale (0 - 10)
Elizabeth L. Westwood et al, 2019 [73]	UK	Cross-sectional study	N=25	56%		Paediatric tracheostomy patients	6,25	Pediatric Quality of Life Inventory Family Impact Module (PedsQL FIM)
Helene Werner et	Switzerland	Cross sectional study	N=126	54,76%	Socio-economic status<br>High 17 (25%)	Children with cardiac	11,2	German version of the Posttraumatic Diagnostic Scale (PDS). German version of the

al, 2019 [74]					Middle 49 (71%)<br>Low 2 (3%)<br>Unknown 1 (1%)	rhythm devices		Medical Outcomes Study Short Form-36 item questionnaire (SF-36)
Pokharel R, et al, 2020 [75]	Nepal	Cross sectional study	Caregivers of Patient with Epilepsy(n=106)	71,7%	Above poverty line 64,2%	Epilepsy	8,6	Zarit Burden Interview short version scale (ZBI-12)<br><br>The Hospital Anxiety Depression Scale (HADS) Nepali version
Taseer F Din et al, 2020 [76]	South Africa	Descriptive observational quality of life assessment. Cross secional study	n=68	60/68-88.2%	19/68-28% full-time employment<br><br>49/68 (72%) before the child's tracheostomy insertion<br><br>44/68 (64%) reported that they would be in employment if they did not have to care for their tracheostomy-dependent child	Children with tracheostomies		The Paediatric Tracheotomy Health Status Instrument . Quality of life carer.
Helen Leonard et al, 2020 [53]	Australia	Cross-sectional study	N=129	86,8%	Mothers Works<br>Homemaker42,6%<br>Part-time 25,6%<br>Full time31%<br>Missing 5,4%<br>Fathers Works<br>Homemaker7,8%<br>Part-time7%<br>Full-time79,8%<br>Missing 5,4%	CDKL5 Deficiency Disorder	11.3	Short form health survey (SF-12)<br><br>Family quality of life was evaluated using the Beach Center Family Quality of Life (BCFQL) Scale

Sang-Ahm Lee et al, 2020 [77]	Korea	Cross-sectional study	N=482	73,7%		Epilepsy	25,5	Stigma Scale–Revised (SS-R)<br><br>The Patient Health Questionnaire-9 (PHQ-9)
Manal M Darwish et al, 2020 [40]	Egypt	Cross sectional study	N=250	100%	Housewife 236 94,4% Working mother 14. 5,6%<br><br>Socioeconomic level. Low 42. 16,8%. Middle 164. 65,6%. High 44. 17,6%	Chronic Kidney disease	11,9	Pediatric Quality of Life Family impact module(PedsQL FIM)
Pablo-A. Conde-Guzón et al, 2020 [78]	Spain	Cross sectional study	N=196 Spain<br><br>N= 219 France			Epilepsy	10,5	EFIQUACEE scale. Parental QoL Difficulties Questionnaire
Filiberto Toledano-Toledano et al, 2020 [35]	Mexico	Cross sectional study	N=416	81,7%	| | Pooled | Mother | Father | Homemaker: 274 (65.9%) | 273 (80.3%) | 1 (1.3%)<br>Student: 3 (0.7%) | 2 (0.6%) | 1 (1.3%)<br>Paid work: 111 (26.7%) * | 54 (15.9%) | 57 (75%)<br>Office employee: 58 (13.9%) | 34 (10%) | 24 (31.6%)<br>Seller/merchant: 39 (9.4%) | 17 (5%) | 22 (28.9%)<br>Laborer: 14 (3.4%) | 3 (0.9%) | 11 (14.5%)<br>Unemployed: 28 (6.7%) * | 11 (3.2%) | 17 (22.4%)	Chronic disease	5,91	The World Health Organization Quality of Life Questionnaire, developed by the WHOQOL Group, Family Functioning Scale, Social Support Networks Scale, Beck Depression Inventory, and The Measurement Scale of Resilience in Mexicans (RESI-M), The Family Support Scale (FSS), *The* Zarit Burden Interview (ZBI), The Parental Stress Scale (PSS), The Well-Being Index of the World Health Organization was developed by Bech et al., The Historical *Socio-*Cultural Premises *Scale* (HSCPS)

Filiberto Toledano Toledano, 2020 [79]	Mexico	Cross-sectional, descriptive, and ex post facto study	N=401	81,3%	Economically remunerated activity Cluster 1 30,84% Cluster 2 24%	Children with chronic disease	Cluster 1 6,04 Cluster 2 5,93	The Beck Depression Inventory BDI-II Second Edition (BDI-II) The Beck Anxiety Inventory (BAI). The Mexican Resilience Measurement Scale (RESI-M) The Zarit Burden Interview (ZBI) Parental Stress Scale (PSS) Family Support Questionnaire (FSQ) The WHO (Ten) Well-Being Index (WHO-TWBI
Kim F. E. van de Loo et al, 2020 [80]	Netherlands	Cross sectional study	N=122	Mothers 110 Fathers 102	Having a job<br>Mothers 65,5%<br>Fathers 91,3%	Mitochondrial Disease	8,3	Parenting Stress index (PSI), Inventory for Social Reliance (ISR)
Sonia Graziano et al, 2020 [81]	Italy	Cross-sectional study	N=186	Mothers 117 62,9%<br><br>Fathers 69	Unemployed N (%) Mothers 23,1% Fathers 1,5%<br><br>Employed Part-Time N (%) Mothers 51,3% Fathers 8,7%<br><br>Employed Full-Time N (%) Mothers 25,6% Fathers88,8%	Cystic Fibrosis	Children 5<br><br>Adolescents 14	Patient Health Questionnaire (PHQ-9)<br><br>Generalized Anxiety Disorder (GAD-7)
Isabelle Werninger et al, 2020 [59]	Switzerland	Prospective cohort study	N=125			Congenital heart diasease	10,17	"Global Severity Index" of the Brief symptom inventory (BSI)
Ammann-Schnell et al. , 2021 [43]	Germany	Case base study/cohort study	Children 49 Parents 43	63,6%	Mothers 60,5%<br>Fathers<br>100%	Leucodistrofia metacromática/Hipoplasia pontocerebel	11,6	Pediatric Quality of Life (PedsQL FIM) Parent HRQOL, Family Functioning Summary Scores.

						osa tipo2		
Magnus Spangsberg Boesen et al, 2021[82]	Denmark	Cohort study	Parents with a chronically ill child (n=13,769)<br>Parents without a chronically ill child (n=138,606)	50%	90%	Multiple sclerosis (n=92)<br>Acute disseminated encephalomyelitis (n=52)<br>Type 1 diabetes mellitus (n=6,191)Inflammatory bowel disease(n=3,388)<br>Rheumatoid arthritis (n=4,685)	11,5	Antidepressant drug redemption or visit to psychologist/psychiatrist clinic.<br><br>Health care utilization<br><br>Employment<br><br>Mortality
Mark A. Ferro et al, 2021 [44]	Canada	Longitudinal study	N=147	89%	Reported annual household incomes of ≥$90,000 (58.9%)<br><br>Completed postsecondary education (78.8%)	Chronic physical illness	9,3	Kessler-6 (K6)<br><br>Center for Epidemiological Studies Depression Scale (CESD), Parental Stress Scale (PSS), and McMaster Family Assessment Device (FAD)
Ryo Morishima et al, 2021[83]	Japan	Cross sectional study	N=125	91,2%		22q11.2 deletion syndrome	11,8	Psychological distress was measured by the Kessler 6 (K6)

Inmaculada Riquelme et al, 2021 [57]	Spain	Cross-sectional study	N=59	One of the parents (mostly the mother), even in divorced families.	Both parents full time employed 62,71% One parent half-time employed 22,03% Unemployed 15,25%	Cerebral palsy	4-18 years	Pediatric Quality of Life Family Impact Module (PedsQLTM 2.0 FIM)<br><br>Pediatric Quality of Life Healthcare Satisfaction Generic Module. (PedsQL HSGM)
A. Strzelczyk et al, 2021 [37]	Germany	Cross-sectional study	N=68	89,7%		Dravet syndrome	10	EuroQol 5D-5L
Kelvin Ying et al, 2021 [48]	Malaysia	Cross sectional study	N=159	79,9%	Less than RM2000(~USD494) 43,4% RM2000-RM3999 (~USD494–988) 28,9% RM4000 (~USD988) and more 27,7%	Cerebral Palsy	0-18<br><br>8-18→60%	Pediatric Quality of Life Inventory Family Impact Module (PedsQL FIM)
Christine Settoon et al, 2021[84]	US	Cross-sectional study	N=26		Full time job (>40 h/week) 25%. Part time job (<40 h/week) 8,3%. In school 8,3%. Unemployed 58,3%	Children with tracheostomies		Pediatric Tracheostomy Health Status Instrument (PTHSI)
Aycan Ünalp et al, 2022 [85]	Turkey	Case–control study	N=60 Epilepsy group<br><br>N=51 Control group			Epilepsy	130,63/136,82 months	The Beck depression scale and Beck anxiety scale (BAI) were used to determining the depres- sion and anxiety levels of the parents.<br><br>Short form-36 quality of life. (SF-36)

Anna Rozensztrauch et al, 2022 [14]	Poland	Cross-sectional survey	N=103	93,20%		Epilepsy	2-25	Pediatric Quality of Life Inventory Family Impact Module (PedsQL FIM)
Merete K. Tschamper et al, 2022 [86]	Norway	Cross sectional study. Cohort	N=254	79,1%	Not working 24,4%<br><br>Working full-time or part-time 75,6%	Epilepsy		The General Perceived Self-Efficacy Scale<br><br>The Hopkins Symptom Checklist-10
Karlie O'Brien et al, 2022[56]	US	Prospective cohort study	N=130 patients undergoing gastrostomy tube placement N=35 patients hernia repair	G-tube 90%<br><br>Hernia 97%	Employed G-tube 49% Hernia 40%<br><br>Not employed G-tube 51% Hernia 60%	Children undergoing gastrostomy tube placement		Pediatric Quality of Life Inventory Family Impact Module (PedsQL FIM)
Maja Brandt et al, 2022 [87]	Germany	Sistematic review	N=24			Spinal Muscular atrophy		Pediatric Quality of Life Inventory Family Impact Module (PedsQL FIM).<br><br>EuroQol EQ5D5L.<br><br>CarerQol.<br><br>Hospital Anxiety and Depression Scale (HADS)

								State anxiety inventory (STAI)<br><br>Zarit Burden Interview.<br><br>Caregiver Strain Index
Vuong Prieto et al, 2022 [33]	US	Cross sectional study	N=32	93,8%	Employed 43,8% Unemployed 56,3%	Children with medical complexity	64,4 month s	Zarit Burden Interview. Caregiving Satisfaction Scale. Short Form Health Survey 12(SF-12)
Cibele Longobardi Cutinhola Elorza et al, 2023 [88]	Brazil	Cross-sectional study. Case-control study	N=29 Cases<br><br>N=53 Control	Cases=86,2%<br><br>Control=81,1 %		Chronic kidney disease	Cases =12,5 Contro l=12,1	The Beck Anxiety Inventory(BAI)<br><br>The Beck Depression Inventory(BDI)<br><br>World Health Organization's Quality of Life Instrument(WHOQOL)
Florencia Epifani et al, 2023 [89]	Spain	Cross-sectional study	N=21			Phosphomannomutase deficiency (PMM2-CDG)	12,4	Parental Stress Index(PSI)
Margarita Maltseva et al, 2023 [90]	Germany	Cross-sectional study	N=108	92,6	Mother's professional situation % (n) Housemaker 28.7 (31) Employed 46.3 (50) Student 1.9 (2) Receiving pension 8.3 (9) Father's professional situation % (n) Housemaker 0.9 (1) Employed 85.2 (92) Student 0.0 (0) Receiving pension 5.5 (6)	Dravet syndrome	13,5	Hospital Anxiety and Depression Scale (HADS).<br><br>Burden Scale for Family Caregivers (BSFC)<br><br>Pittsburgh Sleeping Quality Index

					Change in mother's professional situation due to child's DS % (n) No change 23.1 (25) Yes, quit her work 33.3 (36) Yes, different job 10.2 (11) Yes, reduction of working hours 26.9 (29)<br><br>Change in father's professional situation due to child's DS % (n) No change 88 (95) Yes, quit his work 0.9 (1) Yes, different job 1.9 (2) Yes, reduction of working hours 2.8 (3)			(PSQI)
Hanna Skro banski et al, 2023 [51]	UK	Cross-sectional study	N=73	95%	Employed (part-time) 2 (5) In education or training 10 (24) Unable to work due to their health 26 (62) Unemployed or looking for work 1 (2) Other 3 (7)	Tuberous sclerosis complex	20	Pediatric Quality of Life Inventory Family Impact Module (PedsQL FIM)<br><br>Hospital Anxiety and Depression Scale (HADS)
Jessica Teicher et al, 2023 [91]	Canada	Qualitative study	N=15	66%	Full time and paid 6<br><br>Part time and paid 5<br><br>Not employed and not looking for work 4	Children with medical complexity	0-2→2<br><br>3-6 →7<br><br>7-9 →3<br><br>10-11→ 3	Semi-structured interview
Kadi A Alhumaidi et al, 2023 [92]	Saudi Arabia	Cross sectional study	N=63	50,8%		Cerebral Palsy		22 questions assessing the family quality of life

You Wu et al , 2023 [50]	China	Cross sectional study	N=288	66%	<20000→98(34%)<br><br>20000→97(33,7%)<br><br>>75000→69(24%) (RMB)	Epilepsy	6,4	Family Burden Scale of Disease, GAD-7, PHQ9, WHO Quality of Life Scale (WHOQOL-BREF), and the PedsQL 4.0 Generic Core Scales (parent-proxy report)
Anna Rozensztrauchet al, 2023 [34]	Poland	Cross sectional study	N=16		62,5% employed. 70% of the families included in the study, 1 of the parents gave up working after their child's diagnosis of MD	Menkes Disease		The Author's Own Questionnaire. PedsQL questionnaire PedsQL FIM, Multidimensional Scale of Perceived Social Support (MSPSS)
Bethlyn Vergo Houlihan et al, 2023 [93]	US	Qualitative study	N=127<br><br>Grupos focales 27			Children with medical complexity		127 families in 27 focus groups nation wide

Author, year	Place of study	Pathology	Aim of study	Questionnaires outcomes	Priorities identified
Catherine Marx et al, 2011 [62]	Brasil	Cerebral Palsy	To evaluate depression, anxiety and excessive daytime sleepiness (EDS) levels in primary caregivers of children with cerebral palsy (CCP) and to trace the re-lationships with their socioeconomic conditions and child neurological characteristics, as compared with caregivers of typical children (CTC).	Control Beck<br>7,5 STAI-T<br>40,0 STAI-E 40,7<br>Epworth 8,5<br><br>Cases Beck<br>12,5 STAI-T 45,6<br>STAI-E 43,0 Epworth<br>85,7	Symptoms of depression and anxiety are more common in the cases.<br><br>Excessive daytime sleepiness was not associated with anxiety and depression symptoms in primary caregivers.<br><br>There was no relationship between children's functional independence and symptoms of anxiety, depression, or excessive daytime sleepiness in caregivers
Kjersti Ramstad, 2012 [31]	Norway	Cerebral Palsy	To explore the contribution of recurrent musculoskeletal pain and mental health to aspects of participation in children with cerebral palsy.	Parent GHQ weighted score 1.0 (0.0–5.0)	In this study, lower satisfaction with caregivers' daily activities was associated with mental health problems, being female, and recurrent musculoskeletal pain.<br><br>Parental mental health problems influence various aspects of participation and activities of daily living.

Jennifer L. Hefner et al, 2013 [32]	US	Ventilator-dependent children	To describe the demographic and health status profile of ventilator-dependent children, to identify the types of unmet needs families caring for a child on a ventilator face, and to determine the correlates of access to care coordination.	Caregiver burden Daily caregiver burden > 16 h→29 23.8% Daily caregiver burden 9–15 h→42 34.4% Daily caregiver burden < 8 h→44 36.0%<br><br>Caregiver depressive disorder<br>Positive screen on the PHQ-2→20 16,4%. Although they were below the cutoff for a diagnosable depressive disorder, more than 40% of caregivers reported that in a 2-week period they felt down/depressed and little interest/ pleasure in life "several days" or more.	Children dependent on ventilators are at greater risk for health problems. They are a vulnerable population due to their chronic and complex disabilities, and they also face more barriers to access.<br><br>25.2% of families care for their children for more than 16 hours a day, and 40% of caregivers reported frequent depressive symptoms. Caregiver burden was quite high.<br><br>Families face barriers to accessing treatment and home care.<br><br>Providing mental health interventions for caregivers and alleviating financial hardship can be important ways to help these families.
M. A. Landolt, 2014 [30]	Swiss	Congenital heart defect	To assess child internalizing behavior problems and maternal mental distress in a sample of children with CHD in the first 3 years after birth		Mothers of children with congenital heart disease (CHD) are at significant risk of experiencing intense and lasting distress. Therefore, it is important to monitor them closely and offer them personalized psychological support if they display clinical levels of distress. In addition to individual support, family interventions that address relationships and dynamics between family members are also recommended.
Maria Esposito et al, 2014 [63]	Italy	Neurofibromatosis 1	To assess the maternal stress levels in a	**PSI-SF domains**<br><br>Cases NF1 PD 33.19 PCDI 21,86 DC	The diagnosis of a chronic illness in a child has a significant impact on other family members. This creates stress in parenting,

			population of school-aged children affected by NF1.	26.14 DEF 12.93 TS 84.53<br><br>**Controls** PD 26.94 PCDI 22.18 DC 22.96 DEF 13.96 TS 65.27	which can lead to difficulties in the parent-child relationship, generating distress in caregivers, triggering difficult characteristics in children, and also producing dysfunctional parent-child interactions.
Alexandra L Quittner, 2014 [27]	US	Cystic Fibrosis	Prevalence of psychological symptoms was evaluated in adolescents and adults with cystic fibrosis (CF) and parent caregivers across nine countries.	**Positive scores (%)**<br><br>HADS-D Mothers 618 (20%) Fathers 173 (18%) CES-D Mothers 1057 (34%) Fathers 240 (25%) Either HADS-D or CES-D Mothers 1165 (37%) Fathers 305 (31%) HADS-A Mothers 1496 (48%) Fathers 343 (36%)	The rates in community samples for both depression and anxiety are two to three times lower than those in our sample (37% of mothers and 31% of fathers reported clinically elevated depression; 30% of patients and 36%–48% of fathers had elevated levels on anxiety scales). For caregivers and children, depression or anxiety in either parent doubled the risk of the child reporting elevated psychological distress. Caregiver variables such as older age, female sex, greater illness severity, and recent changes in health status, such as hemoptysis or intravenous antibiotic prescription, were associated with higher depression and anxiety. The results of this study highlight the importance of measuring and intervening in mental health problems in patients and families coping with serious chronic illnesses.

Katarzyna Wojtas et al, 2014 [64]	Poland	Epilepsy	To evaluate the severity of negative emotions in parents of children with epilepsy and ways to deal with the stress in relation to the received social support.	**HADS-M** = Anxiety 8.25 points. Depression 6.02. Higher levels of anxiety and aggression were observed in mothers.<br><br>**Mini-Cope Inventory** = Women reported the following symptoms more frequently than men: "turning to religion," "denial," and "relieving." Men more frequently used "psychoactive substances."<br><br>**Berlin Social Support Scale**: protective support; higher scores were observed in men. Correlation between the level of intensity of negative emotions and social support. Correlation between social support and selected stress-coping strategies presented by the mothers and fathers group.	The results of the study confirmed the presence of depressive symptoms and also revealed high levels of anxiety. These anxiety symptoms were more severe in the group of mothers with higher levels of aggression. Fathers who reported greater satisfaction with care and perceived support had lower levels of anxiety and depression. Social support is a fundamental element in coping with the stress of childhood illness. The study confirmed that, for families, seeking information, feeling understood by the system, and receiving moral support facilitated obtaining social support and receiving help, and this also gave them the feeling that they would continue to receive this support and help in the future.
Xolyanetzin Montero Pardo et al, 2015 [16]	Mexico	Cancer	To identify the psychological characteristics and care variables that predict the onset of primary informal caregivers burden (CPI) of children with cáncer.	**Zarit.** Absence of load 26%. Light load 46% Excessive load 28%<br><br>**Beck Anxiety**. Minimal 20% Mild 34% Moderate 30% Severe 16%. Depression. Minimal 6% Mild 28% Moderate 46% Severe 20%<br><br>**Coping styles Lazzaus and Folkman**<br>Problem-directed 26% Magical thinking 31% Distancing 29% Seeking social support 31% Turning to the positive 27% Level of support No support 28% Medium-low support 32% Medium-high support 13% High support 28%	Most of the caregivers participating in this study experienced some level of burden and a decreased quality of life. These changes can impact the care they provide and their own health.It is important to design psychotherapeutic intervention programs that reduce the emotional burden of a cancer diagnosis in a child and the caregivers' vulnerability to becoming potential patients. The quality of life of pediatric cancer patients is related to the emotional state of their caregivers.

Mark A.Ferro et al, 2015 [65]	Canada	Chronic physical illness	To test a stress-generation model of putative mechanisms that may link chronic physical illness to symptoms of anxiety and depression in a population- based sample of children aged 10-15 years.	Depressive symptoms<br><br>CPI→5,1<br><br>H→4<br><br>Family dysfunction<br><br>CPI→8,4<br><br>H→8,2	The study assesses the possible relationship between chronic physical illnesses, maternal depressive symptoms, family dysfunction, and children's self-esteem and anxiety and depression symptoms.<br><br>For mothers, the diagnosis of chronic illnesses in children has negative consequences that can lead to impaired family functioning. These adverse consequences include increased care needs, higher treatment costs, anticipated grief, feelings of stigma, and higher levels of depression.<br><br>The study finds that anxiety and depression symptoms in children with chronic illnesses are influenced by the consequences of chronic illness diagnoses, such as elevated levels of maternal depression, family dysfunction, and low levels of self-esteem in children.
Rodrigo López et al, 2015 [58]	Chile	Congenit Heart Disease	to assess the effect of medical treatment on well-being and agency in parents of children having CHDs, in Chile, and to compare it with reference values.	BPN Control 66,2 Cases preop 66,18 post 64,80<br><br>SDS Control 40,39 Cases preop41,71 post 41,90<br><br>Hopelessness Control 2,58 Cases preop 3,313 post 2,18<br><br>GHQ Control 9,21 Cases preop13,82	Childhood illness was associated with lower levels of quality of life. Childhood surgery improved caregivers' hope. Fathers reported greater perceptions of autonomy and competence than mothers before and after surgery. Low socioeconomic levels were associated with worse outcomes on the health questionnaire. Healthcare systems must address the well-being of families when children have chronic illnesses, placing special emphasis on those who have secondary burdens, such as those from low

				post 12,44	socioeconomic levels or situations of special family vulnerability due to sex or gender.
Sarah Johaningsmeir et al, 2015 [5]	US	Children with medical complexity	To measure longitudinal changes in quality of life and health care satisfaction of family caregiver of CMC over 2-year period following enrollment in complex care program	Peds QL FIM. Social Functioning 56.25,50,50. Worry 50,45,50. Daily Activities 50, 50, 41.67.<br><br>PedsQL HCS relatively high compared FIM: . The lowest score were in the Information and Emotional Needs domains.	Family caregivers of children with medical complexity and multiple conditions and high resources had low scores on the PedsQL FIM scale from the start of care in a complex care program and showed no change in the following two years. Caregivers' PedsQL HCS scores were higher at baseline and also did not change over time. The results suggest that caregivers of children with medical complexity have low levels of quality of life but do not identify causes or solutions.
Ahmad Azhar et al, 2016 [29]	Saudi Arabia	Congenital Heart Diseases<br>Simple 69,5%<br>Complex 30,5%	To assess the impact of CHD on the bio-psychosocial aspects of the QOL of both patients and their family members, including siblings.	18.9% of mothers and 9.4% of fathers had difficulty coping with their child's illness.<br>Self-reported depression was three times higher in mothers than in fathers.<br>32.8% of siblings felt jealousy toward their sick brother or sister, and 19.4% felt neglected by their parents because of the illness.	No impact on the caregivers' quality of life was observed due to the severity of the congenital heart disease.<br>A higher percentage of the mothers of caregivers who were exclusively dedicated to caregiving experienced a significant impact on their quality of life. 34% of mothers in this group gave up important social activities to care for their children.<br>The inclusion of spiritual support improves the management of these patients and their families.<br>The mothers experienced a similar impact as the children.
Bahar Büyükkaragöz et al, 2016 [66]	Turkey	Renal transplant	Evaluates the health-related quality of life (HRQoL) of	SF-36<br>PHS45,4 42,9<br>MHS 46,7…45,73	Pediatric patients and their caregivers have an adequate quality of life.

			pediatric renal transplant recipients and their parent.	RSES 0,60…0,14<br><br>BDI 9,58…11,83	Due to having to cope with all the caregiving duties for their children and the fear of organ rejection, caregivers tended to become depressed.<br><br>To improve the quality of life for both families and children in the post-transplant period, it is necessary to include ongoing education and counseling.
Ozalp Ekinci et al, 2016 [28]	Turkey	Epilepsy	Compare sleep problems between children and adolescents with epilepsy and non-epileptic controls, and (2) examine whether there is an association between sleep problems and quality of life, ADHD and mothers' emotional symptoms.	STAI state- CSHQ <56(n=35)=44,58<br><br>CSHQ >56(n=18)=44,11<br><br>STAI trait- CSHQ <56(n=35)=47,38<br><br>CSHQ >56(n=18)=52,44<br><br>Beck- CSHQ <56(n=35)=8,51<br><br>CSHQ >56(n=18)=14,55	Mothers of children with epilepsy are at increased risk for anxiety and depression symptoms due to the chronic burden of the disease, the unpredictable nature of seizures, and the associated social stigma.<br><br>The relationship between epilepsy and sleep problems in children makes it necessary to assess the comorbidity of epilepsy with ADHD, and it is also essential to evaluate maternal emotional symptoms.
Adeniran O. Okewole et al, 2016 [67]	Nigeria	Neuropsychiatric disorders<br>63% seizure disorders<br>40%comorbid diagnosis	To investigate the relationship between maternal depression and child psychopathology among attendees at a specialist child and	Single mothers were more likely to screen positive for major depressive disorder. Children of mothers with major depressive disorder had a significantly longer life expectancy. Maternal depression is related to the mothers' marital status, the child's primary diagnosis, and the duration of the illness. Mothers of children with intellectual disabilities or other disorders had higher rates of depressive disorders compared to mothers	High levels of depression were found in mothers of children with neuropsychiatric disorders. High levels of emotional and behavioral disorders were also found in the children. There was a direct association between psychopathology in the mothers and children.

			adolescent mental health facility in Nigeria.	of children with seizure disorders.	
Soyong Eom et al, 2017 [54]	Korea	Pediatric Mitochondrial Disease	To delineate neurodevelopment and psychological comorbidity in children with mitochondrial diseases in the preliminary investigation of adequate intervention methods, better prognoses, and improved QOL for both patients and parents.	Parenting Stress: K-PSI 88.6<br><br>Maternal Depression: BDI 14.6	High levels of parental stress were found.<br>The overall stress in the parent-child system is influenced by the child's characteristics.<br>Children with mitochondrial diseases require a great deal of care and demand parental participation, and parents are particularly affected by both the caregiving demands and disappointment in their children's overall adaptive functioning. Parents have greater difficulty bonding with their children, which leads to decreased parenting competence and ongoing distress due to the lack of attachment, which would hinder family functioning.<br>Sixty-five percent of mothers had high levels of depression.
Krista Keilty et al, 2017 [68]	Canada	Children who depend on medical technology (CMT)	To compare sleep in family caregivers of CMT with that of family caregivers of healthy, same-	Total sleep time CMT 6,56 Cobtrol 7,21<br>PSQI CMT 7,75 Control 5,45<br>SF-12 physical CMT41,81 Control 42,18<br>SF-12 mental CMT 47,25 Control 49,81<br>CES-D CMT 11,7 Control 6,95<br>CES-D 16-21 CMT 7 CONTROL 3<br>CES-D >21 CMT 5 CONTROL 1<br>Epworth Sleepiness Scale CMT 8,35	This study found that family caregivers of children with CMT do not get enough sleep and are at risk for sleep-related problems such as depression, daytime sleepiness, and fatigue. Variability in sleep duration is also linked to these symptoms, which may affect

			aged children.	Control 5,07 ESS >10 CMT 16 Control 5 Multid. Assement of fatigue CMT 22,12 Control 17,44	their motivation and ability to care. These caregivers were found to tend to have lower socioeconomic status. Health-related quality of life was not affected by sleep disturbances.
Nicole Villas et al, 2017 [38]	US	Dravet syndrom	to (1) identify top concerns among caregivers, (2) establish an approximate frequency of characteristics and comorbidities of DS beyond seizures, and (3) provide direction for clinicians and researchers looking to study the effects of DS on the patient and family unit.	Sixty-six percent of caregivers reported experiencing depression, and only 26% had received some form of family therapy. Seventy-four percent of caregivers reported concerns about the emotional impact on siblings, which also appeared as a common concern in the open-ended section of the survey. They were asked to rank their top three concerns in an open-ended response: seizure control, behavioral and communication disorders, and the impact on the family of needing to care for a child with Dravet syndrome.	Most caregivers, 80%, sleep with their children for safety, and half use a pulse oximeter, likely due to fear of sudden death in epilepsy (SUDEP). This generates stress, depression, and vulnerability in the family. Dravet syndrome primarily affects the patient, but also caregivers and the entire family. Caregivers believe that treatment should go beyond seizure medications, also including attention to behavioral, developmental, and communication issues, and providing support for caregivers.
Klajdi Puka et al, 2018 [39]	Canada	Epilepsy	Identify the prevalence of depressive symptoms among mothers of CWE at the time of diagnosis and 0.5, 1, 2, 8, and 10 years later; identify	Mothers' mean CES-D score was 14.5 (SD = 10.5) at baseline, 11.6 (SD = 9.5) at 6 months, 12.2 (SD = 9.7) at 1 year, 11.9 (SD = 10) at 2 years, 11.2 (SD = 9.6) at 8 years, and 9.8 (SD = 9.0) at 10 years<br><br>Low-Stable 29% Intermediate-Stable 46% High-Stable 20% High-	Over the course of 10 years, depressive symptoms in parents remained stable, even when seizures improved. Fifty-seven percent of mothers of children with epilepsy faced a risk of major depression at least once during that period. Furthermore, a positive family environment at the time of diagnosis was associated with better long-term outcomes from depressive

			trajectories of mater- nal depressive symptoms; and identify the baseline child, maternal, and family characteristics associated with depressive symptom trajectories.	Decreasing 5%;<br><br>Resources-FIRM-At diagnosis 50,1 Follow 10y 52(+)<br><br>Demands-FILE- At diagnosis 9,5 Follow 10y 8,1(-)<br><br>Functioning APGAR- At diagnosis 13,9 Follow 10y 14,8.(+)	symptoms. These findings demonstrate the importance of paying special attention to parents' mental health through family-centered interventions.
Klajdi Puka et al, 2019 [69]	Canada	Epilepsy	To 1) describe the quality of life (QOL) of parents of children with childhood-onset epilepsy (CWE), 2) identify factors associated with parental QOL, and 3) evaluate the association between parents' QOL and children's psychological well-being.	Parents of CWE had worse mental health-related quality of life relative to population norms or control groups. Six studies reported that parents of CWE had similar or marginally better physical health-related quality of life relative to controls; however, two studies reported lower scores on some subscales related to physical health-related quality of life and found that the quality of life of parents of CWE was better relative to the quality of life of parents of children with obesity and similar to the quality of life of parents of children with asthma, diabetes, and cerebral palsy. Similar quality of life was found between parents of CWE and parents of children with brain tumors. Parents of CWE (those with CDKL5) had poor mental health-related quality of life but better physical health-related quality of life relative to parents of children with Down syndrome and Rett syndrome. Studies consistently found that higher child quality of life, higher parental anxiety and depressive symptoms, and lower socioeconomic status were associated with poorer parental quality of life.	Parents of children with epilepsy (CWE) often have lower scores across most quality of life domains, particularly mental health. Influential factors have been identified, and the importance of further research is highlighted to understand how these factors, such as family environment, psychosocial factors, coping strategies, and management behaviors, influence parents' quality of life and, consequently, that of their children. Evidence shows that parents' psychological well-being and a healthy family environment are closely related to children's mental health and well-being. Therefore, it is essential to consider the impact on the entire family.

Yuca Mori et al, 2018[55]	Australia	Rett syndrome	To examine longitudinally the well-being of parents of individuals included in the Australian Rett Syndrome Database over the period from 2002 to 2011.	**Short Form 12 Health Survey**<br><br>PCS 46.6, 47.1, 46.1, 47.3<br><br>MCS 46.4, 45.5, 47.3, 45.6<br><br>**Family function**<br><br>Better 46.2, 49.3%, 56.1%, 51.6%<br>Poorer 50%, 49.3%, 42.7%, 47.8%	Physical well-being deteriorated over the 9-year follow-up in both the general population and the children's families. The general population had slightly higher emotional well-being than caregivers. Parents found strategies and resources to cope with the burden of Rett syndrome. Factors affecting parents' physical and emotional well-being included distance from healthcare facilities, the child's early age, the type of MECP2 gene mutation, sleep disturbances, and the child's behavioral problems. Caregivers' physical well-being was related to family function and parental income, and emotional well-being was associated with difficulties feeding the child. Caregivers' physical and emotional well-being was better when the child lived in foster care.
Adam Strzelczyk et al, 2019[70]	Germany	Dravet syndrome		EQ-5D-3L 0.9 (0.18)/ EQ-VAS 71.3 (18) not differ from the general German population.<br><br>Forty-five percent of carers scored >13 points on the BDI-II, indicating symptoms of mild (22%, n 1⁄4 20), moderate (15%, n 1⁄4 14) or severe (9%, n 1⁄4 8) clinical depression .<br><br>Total mean annual cost per DS patient of 45,824E or 51,525E	The financial resources required by families of children with Dravet Syndrome(DS) are significant and depend on the severity of comorbidities. Caregivers of children with epilepsy reported a high frequency of depressive symptoms (45%). EQ-5D-3L and EQ-VAS scores for caregivers of children with DS were similar to those of the general population. This suggests that tools that measure only health-related quality of life may not fully reflect the true impact that DS has on caregivers.

Ellison Suthoff et al, 2019 [71]	Germany, Ireland , United Kingdom, and the US	Cystic Fibrosis	To describe the burden of pulmonary exacerbations experienced by primary care-givers of children with cystic fibrosis.	SF-12 mental health was affected by pulmonary exacerbation-related hospitalization events 39.8 (10.9) at T1 vs 42.5 (10.4) at T2 .Physical health 52,7-52,4<br><br>WPAI: Caregivers reported a significant increase in burden due to caring for a child with CF in the previous 7 days in the absen- teeism (mean [SD] increase, 30.1% [28.8%]and presenteeism (22.2% [34.6%].Leading to a mean work productivity loss of 32.8% (37.8%). The mean (SD) increase in activity impairment across all caregivers at T1 vs T2 was 23.6% (37.3%).Employed caregivers reported an increase in missed hours at work, with a mean (SD) of 16.7 (14.7).<br><br>CQOLCF 59,6-57,3<br><br>MSPSS. Social support hight 5,3-5,1˙ Family support was significantly increased at T1 relative to T2 5,3-5,0<br><br>CHQ-PF28. Caregivers reported a significantly greater impact on emotional worry/concern, greater limitation in time available for personal needs due to the child's physical health and emotional well- being, and greater interruption in family activities at T1 vs T2.	The variable that places the greatest burden on primary caregivers of children with cystic fibrosis is pulmonary exacerbations requiring hospitalization.
Mayra L. Almanza-	Canada	Epilepsy	To assess whether patient, caregiver,	Anxiety symptoms, GAD-7 score 6,80	More than a third of children (37.2%) have unmet medical

Sepulveda, 2019 [45]			and family factors correlate with unmet healthcare needs in children with drug-resistant epilepsy (DRE) who were evaluated for epilepsy surgery.	Depressive symptoms, QIDS score 6,93<br><br><u>Family characteristics</u><br>FILE, mean 371,05<br>FIRM, mean 49,18<br>APGAR, mean 7,10	needs, especially in the physical (24.4%) and mental (15.1%) areas. Furthermore, 20.9% of caregivers show symptoms of moderate to severe depression, and nearly 28% have symptoms of severe anxiety. Unemployed caregivers are more likely to perceive unmet needs for their children compared to employed caregivers. However, factors related to caregivers or the family, such as family relationships, resources, or demands, do not appear to predict these unmet needs.
Margot A. Lazow, 2019 [72]	US	Osteogenesis Imperfecta	To evaluate stress, depressive symptoms, and quality of life (QOL) among caregivers of children with osteogenesis imperfecta (OI) and to determine if associations exist with patient disease-related characteristics.	PIP Total Frequency M = 97.73 PIP Total Difficulty M = 87.02 FIM Total M = 70.08 FIM Parent HRQOL M = 71.14 FIM Family Functioning M = 72.35 CES-D M = 8.16	Caregivers of children with osteogenesis imperfecta (OI) who have patients with more pain and lower physical functioning experience more stress and a poorer quality of life. However, the stress level and quality of life of these caregivers is similar to that of caregivers of children with other chronic illnesses, but worse than that of parents of healthy children. The frequency of depressive symptoms in these caregivers is similar to that of the general adult population. These characteristics can help identify caregivers at risk. Furthermore, collaboration between physicians, nurses, social workers, and administrators is key to recognizing these symptoms, providing appropriate resources, and implementing targeted interventions.
Fadwa M. S. Mohammed et al	Sudan	Cerebral Palsy	To measure the QoL of CP	Caregivers QoL	The quality of life of both children with cerebral palsy and their caregivers is low.

,2019 [47]			patients and their caregivers and determine the factors a ecting both of them	Overall QoL 8,85<br>Physical QoL 2,00<br>Social QoL 2,18<br>Support for caring 3,48<br>Financial burden 1,1	Socioeconomic status, which is related to occupation, influences this quality of life. Furthermore, factors such as parents' knowledge about cerebral palsy and their educational level are also important; the higher the level of education, the better the quality of life for the caregiver. To improve this situation, it is recommended to pay more attention to families in care planning and to include a psychotherapist on the care team to strengthen parents' confidence and coping strategies.
Dominique L. Denniss et al, 2019 [52]	Australia	Complex Congenital Heart Disease.<br>Single ventricle group(n=30)<br>Biventricular group (n=57)	To assess health-related quality of life (HRQOL) in families of young children with complex congenital heart disease (CHD), and identify the demographic, clinical, and psychosocial factors that place these children and their mothers at greater risk of vulnerability	**Total Family Impact** 72.16 Single ventricle group 63.61 Biventricular group 76.43 **Maternal HRQOL** 76.43 Single ventricle group 64.87 Biventricular group 75,38 **Family Functioning 76,45** Single ventricle group 67,53 Biventricular group 80,92 **DASS21 (0-21)** Moderate to severe symptoms of depression, anxiety, and stress were reported by 14%, 9%, and 12% of mothers, respectively.	Variables such as poorer family functioning, greater maternal psychological comorbidity, child health problems, and difficult childhood temperament are strongly associated with lower health-related quality of life (HRQoL) in mothers. The relationship between maternal psychological stress and children's emotional, behavioral, and neurocognitive outcomes is well established and likely influenced by biological and environmental factors. This supports the idea that children's clinical factors, rather than psychosocial factors, are important predictors of maternal HRQoL. Therefore, strengthening parents' psychological adjustment and family functioning could improve mothers' quality of life.
Akanksha Rani et	India	Epilepsy	To understand the stress and	Parents of children with epilepsy and	Demographic factors such as being younger, having a lower educational level, and a low

al, 2019 [41]			perceived stigma among parents of children with epilepsy and to find out the association between parental stress and perceived stigma.	co-morbid condition<br><br>Parental stress (frequency) 130.50<br>Parental stress, (difficulty) 131.4<br>Perceived stigma 21.90<br><br>Parents of children with epilepsy without co-morbid condition<br>Parental stress (frequency) 110,43<br>Parental stress, (difficulty) 111.4<br>Perceived stigma 19,6	socioeconomic status are associated with greater stress in parents. Children with conditions such as cerebral palsy, developmental delay, or epilepsy require assistance with daily activities such as feeding, bathing, or communicating, which increases their dependence on parents and, consequently, their stress. Furthermore, many of these children have not yet started school or have dropped out due to seizures, leaving parents feeling helpless and sad about the impact on their children's lives. They also face difficulties communicating with healthcare professionals and need emotional support from the medical team to better cope with the situation.
Janna Riechmann et al,2019 [46]	Germany	Epilepsy	(1) Does QoL in children and adolescents correlate with different socio-demographical and disease specific aspects? (2) Does QoL in caregivers correlate with different sociodemographical and disease	EQ-5D 20-29 years 85,1 ; 30- 39 years 76.1; 40-49 years 76,3; 50-59 years 77; 60-69 years 66.<br><br>BDI-II 28.1% (n=133) of caregivers, a BDI-II score of 13 points or more representing suspected depression was calculated, with 85 cases (18.0%) having mild, 37 cases (7.8%) having moderate, and 11 cases (2.3%) having severe symptoms of depression respectively.	Caregivers of children with epilepsy showed a significantly poorer quality of life compared to healthy individuals, especially on the visual analog scale of the EQ-5D questionnaire. Factors such as having non-idiopathic epilepsy, not having seizures at the time, having other illnesses, significant disability, episodes of status epilepticus, and the primary caregiver being unemployed increase the risk of poorer QOL and are also related to symptoms of depression in caregivers. The longer the disease lasts, the worse the caregiver's quality of life, although this does not affect the

			specific as- pects? (3) Does QoL children and adolescents reciprocally correlate with QoL in their caregivers?		patients themselves as much. Furthermore, the quality of life of children and adolescents with epilepsy is positively related to that of their caregivers, and there is also a significant negative correlation between the patients' quality of life and the caregivers' depressive symptoms. In total, approximately 28% of caregivers presented symptoms of depression. A strong impact of epilepsy on the emotional state of caregivers is evident from the levels of anxiety and depression they report.
Filiberto Toledano-Toledano et al, 2019 [36]	Mexico	Children with chronic diseases	To identify multivariate sociodemographic and psychosocial variables as well as sociocultural and familial factors to analyze the caregiver burden of family caregivers of children with chronic dis- eases.	**Sociocultural premises** →Woman 49,30 Men 50,21<br><br>**Social support networks**→Woman 159,63 Men 156,23<br><br>**Family support** →Woman 59,50 Men 59,21<br><br>**Family functioning**→Woman 86,82 Men 84,43<br><br>**Well-being**→ Woman 18,13 Men 18,55<br><br>**Stressors**→Woman 12,72 Men 12,89<br><br>**Anxiety**→Woman 14,74 Men 13,56<br><br>**Caregiver burden**→Woman 23,12 Men 23,12	Psychological variables influenced caregiver burden. Adequate family functioning is associated with lower perceived burden. Family support is a factor that contributes to positive adaptation during diagnosis and treatment in children and their families, and which could be considered the strength with the greatest impact on caregivers. This study highlights the importance of understanding caregiver profiles through profiling, considering psychosocial and sociodemographic aspects. This is essential for improving research on families with children with chronic illnesses, as it helps design measurement, evaluation, and intervention programs. The goal is to reduce the impact of the illness, its emotional and social effects, as well as the burnout faced by caregivers.

, Oscar Werner et al, 2019 [42]	France	Congenit Heart Disease	To analyze parental anxiety using a visual analog scale and to explore the influencing factors.	Parental anxiety 7,6<br><br>Fathers 6,9 Mothers 8,2	Mothers' anxiety was significantly higher than fathers' anxiety. Paternal anxiety, child comorbidity, distance from home to referral center, and level of adjustment for congenital heart surgery risk were associated with high levels of maternal anxiety.
Elizabeth L. Westwood et al, 2019 [73]	UK	Paediatric tracheostomy patients	(1) To better outline quality of life outcomes in paediatric tracheostomy patients and their caregivers. (2) To establish whether quality of life outcomes for children are associated with those of their caregivers. (3) To establish whether quality of life outcomes were associated with the demographic and clinical variables of age, duration of time with a tracheostomy, number of comorbidities, and ventilator dependence.	Mean caregiver HRQoL was 63.8 (19.8), Family Functioning Summary Score (daily activities and family relationships) was 68.1 (19.7) and total Family Impact Score (physical, emotional, social and role functioning, communication, worries, family relation- ships and daily activities) 61.6 (18.6) Correlations between Patient Psychosocial Health Summary Scores and both Caregiver HRQoL and Caregiver Total Family Impact Score	The burden faced by children with tracheostomies and their families is especially high, even more so than with other serious childhood illnesses. Caregivers feel they do not have enough time or energy for their daily activities, face social isolation, constant worry and difficulty communicating, and feel they do not fully understand their children's health. All of this significantly reduces their quality of life. Furthermore, most caregivers are very worried about the future, which causes anxiety and affects their emotional well-being.
Helene Werner et al, 2019 [74]	Switzerland	Children with cardiac rhythm devices	To assess the prevalence of PTSD in the parents of children with PM or ICD. (2) to describe HRQoL in the patients' parents as compared to parents of sex- and age-	Six mothers (13%) and 4 fathers (10%) met the criteria for full PTSD (due to any traumatic event). The child's heart disease was stated as a single and/or the most troubling traumatic event by 8 mothers (12%) and 4 fathers (6%). Of these, one mother (1%) met the criteria for full PTSD and 3 mothers (4%) for	Mothers of children with heart disease reported significantly lower quality of life scores in almost all areas except "emotional role," compared to a control group. Fathers, on the other hand, only showed a decline in "mental health." The greatest difference between parents and controls was found in "mental health," with both groups

			matched healthy controls and to analyze maternal and paternal differences. (3) to examine associations between parental HRQoL and child medical characteristics as well as associations between parental HRQoL and post-traumatic stress symptoms, while controlling for child medical characteristics and parental socio-demographic characteristics.	partial PTSD. None of the fathers met the criteria for full PTSD diagnosis due to their child's heart disease, while two fathers (4%) met the criteria for partial PTSD.<br><br>PCS Patients Mothers 54.7 Fathers 53.9. Control Mothers 56,8 Fathers 54,8<br><br>MCS Patients Mothers 48.7 Fathers 49.5. Control Mothers 52,1 Fathers 53,2	of parents reporting worse mental health than parents in the control group. Furthermore, some mothers and fathers experienced post-traumatic stress disorder (PTSD) related to their children's heart disease, especially in cases of complete heart disease. The severity of parents' PTSD symptoms was a significant predictor of their mental health, even after adjusting for other factors such child age at implantation, presence of other non-cardiac disease in the child, parental age, and presence of own chronic disease.
Pokharel R, et al, 2020 [75]	Nepal	Epilepsy	This study is expected to assess burden and its predictors among caregivers of children with epilepsy.	ZBI-12 scoring, 59% caregivers had no to mild burden, 27% had mild to moderate burden and 14% had high burden.<br><br>Borderline anxiety 7.5% Borderline depression 8.5% Anxiety 6.6% Depression 6,6%. Borderline anxiety and borderline depression 5.6%<br>Anxiety and depression 3.7%	No conclusive association was found between workload and education.<br><br>Intellectual disability and cerebral palsy were the most common comorbidities.
Taseer F Din et al, 2020 [76]	South Africa	Children with tracheostomies	To assess the QOL of tracheostomised children and their families in a	Quality of life of the carer Max score 85// 62,3- 73,3%// Rango 29-83	The Breatheasy© program helps children and their families live more independently from the hospital, giving them the confidence to thrive. The decision to perform a tracheostomy on a child should

			LMIC using the Paediatric Tracheotomy Health Status Instrument (PTHSI). as the first study of its kind in a low-resource setting .		not depend on the caregiver's education level, their economic situation, or whether they live in formal or informal housing. Children with comorbidities require more support, and caring for a tracheostomy at home can worsen the socioeconomic situation in families who may already be struggling with this. Furthermore, many caregivers would be working if they didn't have to care for their child with a tracheostomy. These families face many additional stresses, and in some cases, children may be less cooperative with their care, requiring more specialized care focused on their needs.
Helen Leonard et al, 2020 [53]	Australia	CDKL5 De ciency Disorder	To describe and investigate factors affecting QOL and the individual domains of QOL in children and adults with CDD.	MentalComponentSummary(MCS) (n=124) 41.9(10.2)(range20.7−63.2)<br><br>Physical Component Summary (PCS)[b] (n =124) 53.6 (7.4) (range 29.3−64.8)<br><br>Family Quality of Life[c] (n=127) 3.3 (0.7) (range 1.8−4.7)	There is a positive correlation between family quality of life and children's quality of life, especially in areas such as positive emotions and total quality of life. When the family has a good quality of life, this can help improve the lives of children with DCD, as these children are highly dependent on family care, support, and resources. Furthermore, having a parent with a full-time job also has a positive effect, especially on their own physical health, and this influences the family's socioeconomic status.
Sang-Ahm Lee et al, 2020 [77]	Korea	Epilepsy	The current study evaluated family members of pediatric and adult patients with	Stigma Scale–Revised, n (%)<br><br>Score = 0→45,9%	Fifty percent of the children's families felt some degree of stigma. Depressive symptoms were significantly related to family stigma. Factors associated with family stigma and depressive symptoms include

			epilepsy and aimed to determine the prevalence of family stigma and depressive symptoms among family members	Score 1–3→44,2%<br><br>Score 4–9 →10%<br><br>PHQ-9 score ≥ 10, n (%)→11,2%<br><br>Comorbidities were noted in 159 (33.0%) of patients with epilepsy (intellectual disability, n = 107; neurological, n = 60; psychi- atric, n = 5; and other medical conditions, n = 28).	frequent seizures, AED combination therapy, comorbidities, and the caregiver's relationship with the patient.
Manal M Darwish et al, 2020 [40]	Egypt	Chronic Kidney disease	To assess family impact and economic burden of CKD in children on their families.	**PedsQL FIM** Parent HRQol summary score 48,8 Family summary score 72,7 Total Score 51,9 (*)<br><br>6.8% of caregivers were completely covered by health.<br><br>Predictors of total PedsQLTM Family Impact<br><br>Responding caregiver<br><br>Degree of financial hardship<br><br>Treatment modality<br><br>Socioeconomic level<br><br>Stage of CKD	Scores on quality of life and family functioning (FIM and PedsQL™) were lower in our population compared to other studies of children with chronic diseases. Mothers and sisters of children with CKD reported greater difficulties, likely because they assume more responsibilities and lifestyle changes. Furthermore, caregivers with greater financial difficulties related to their child's CKD had lower scores in all domains assessed. Family cooperation and cohesion are common characteristics for coping with difficulties. Children with CKD for more than 5 years reported better family quality of life than those with a shorter period of disease. Also, caregivers of children receiving conservative treatment (rather than hemodialysis) and those with higher socioeconomic status reported better scores on quality of life and family functioning.

Pablo-A. Conde-Guzón et al, 2020 [78]	Spain	Epilepsy	To study QoL in two large samples of children with epilepsy from two different countries using this QoL scale in order to analyze the influence of medical factors (age of onset, type of epileptic syndrome or treatment).	**Parent Qol Dificultties**<br><br>France 6,5<br><br>Spain 3,7<br><br>Maximum score = 13	In both countries, the children's illness, especially non-idiopathic and unclassified generalized epilepsies, had a significant impact on parents' lives, with parents reporting higher levels of anxiety, sleep problems, and feeling less calm. This impact was significant regardless of the specific pathology. Furthermore, in general, French parents reported that the illness affected their quality of life more than Spanish parents.
Filiberto Toledano-Toledano et al, 2020 [35]	Mexico	Chronic diseases	To identify the predictors of the QOL of parents of children with chronic diseases	WHOQOL Questionnaire 83,78<br><br>Family Support Scale 59.44<br><br>Family Functioning Scale 69.47<br><br>Social Support Networks Scale 159.01<br>Mexican Resilience Measurement Scale 133.75<br><br>Well-Being Index 18.20<br>Beck Depression Inventory 13.81	In this sample of parents of hospitalized children with chronic illnesses, it was found that having a secondary or tertiary education, older age (around 32 years), and a monthly household income above USD 279.36 were associated with higher levels of parental well-being (QoL). Furthermore, higher levels of resilience, fewer depressive symptoms, more social support, and better family functioning also contributed to parents' greater well-being. No parents reported very low levels of well-being. Finally, it is suggested that parents with lower educational levels could benefit from group therapies focused on strengthening resilience.

				Zarit Burden Interview 23.2<br>Parental Stress Scale 19,68<br>HSCPS 49,47			
Filiberto Toledano Toledano, 2020 [79]	Mexico	Children with chronic disease	To identify the psychosocial profiles of family caregivers of children with chronic diseases and to describe the relationship between these profiles and sociodemographic variables.	Anxiety<br>Caregiver burden<br>Family support<br>Depression<br>Resilience<br>Parental stress<br>Wellness	20.55<br>29<br>56.15<br>18.56<br>126.13<br>22.11<br>15.54	6.74<br>15.56<br>63.86<br>8.41<br>142.46<br>16.68<br>21.26	Two profiles of family caregivers of children with chronic illnesses were identified. Profile 1, called "Vulnerability of family caregivers," includes caregivers with high levels of anxiety, depression, stress, and burden, but with low levels of family support, resilience, and well-being. This profile indicates that these caregivers are at risk of developing psychological problems that can affect the quality of care they provide. Profile 2, called "Adversity of family caregivers," shows caregivers with high levels of support, resilience, and well-being, and low levels of anxiety, depression, and stress, which contribute to improving the child's care and health. Although the sociodemographic characteristics are similar in both profiles, the main difference lies in the type of family (single-parent or nuclear). In summary, the psychosocial profile of caregivers can be organized according to these characteristics to better understand their needs

					and how they impact the child's care and health.
Kim F. E. van de Loo et al, 2020 [80]	Netherlands	Mitochondrial disease	To investigate the psychological well- being of children who are suspected for MD and their parents.	PSI Mothers 47,80 Fathers 49,10<br>IRS Mothers 17,01 Fathers 16,82	Both fathers and mothers in the clinical population reported less parental stress compared to the non-clinical population. Furthermore, only a small percentage of mothers (5.9%) and fathers (11.1%) experienced stress levels in the clinical range. Surprisingly, most fathers did not report high levels of stress, and mothers even reported less stress compared to the general population. The study also found no differences in parental stress between parents of children with or without a diagnosis, as it focused on stress before the diagnosis was made.
Sonia Graziano et al, 2020 [81]	Italy	Cystic Fibrosis	To: a) evaluate the prevalence of depression and anxiety in an Italian sample of adolescents and adults with CF and parent caregivers, using the recommended tools; b) report the comorbidity of depressive and anxious symptoms; c) examine the concordance between psychological symptomatology in adolescents with CF and their parent caregivers; and d) investigate the association between these psychological symptoms and physical health	PHQ-9 mean (SD) Mothers 6,7 Fathers 5,5<br><br>GAD-7 mean (SD) Mohters 5,0 Fathers 6,4	The study shows that many parents who care for their children experience symptoms of anxiety and depression, with a large percentage at moderate to severe levels. Approximately one-third of parents experience depression and half experience anxiety. Furthermore, those with elevated symptoms in one of these areas often also have elevated symptoms in the other. Parents with higher levels of anxiety also have adolescent children who report increased anxiety. Rates of symptoms among patients who attended the Cystic Fibrosis Center in Rome are two to three times higher than normative data. Finally, a significant percentage of adolescents and mothers reported suicidal ideation, 11% among adolescents and 7% among mothers.

			outcomes.		
Isabelle Werninger et al, 2020 [59]	Switzerland	Congenital Heart Disease	To explored all, medical, psychological, and social risk factors, including maternal mental health, as predictors for adverse behavioral outcomes.	Maternal mental health measured with the BSI was in the normal range with Mean T-score = 47.13 (SD = 12.21, range = 21–80; higher scores indicate more problems).	The study found that a child's intellectual abilities and the mother's mental health during early childhood are factors that predict the child's behavior at age 10. In other words, parents' mental health at this early stage influences aspects such as social interaction and ADHD symptoms, even years later. These results highlight the importance of early identification of families at risk for mental health problems in order to offer them the necessary support and promote healthy development.
Ammann-Schnell et al. , 2021 [43]	Germany	Leucodistrofia metacromática/Hipoplasia pontocerebelosa tipo2	To investigate the impact of these diseases on the affected families, e.g. mothers, fathers and non-affected siblings	MLD and PCH2 families reported significantly poorer HRQOL than the families with healthy children in the overall domains of Total Impact Score, Parent HRQOL and Family Functioning Summary Scores	The time elapsed until diagnosis was a great burden for families.<br>A very distressing period occurred in the final stages of the disease, when severe symptoms manifested.<br>The child's illness had a significant negative impact on the family's quality of life.<br>Mothers had lower HRQoL.
Magnus Spangsberg Boesen et al, 2021[82]	Denmark	Multiple sclerosis (n=92) Acute disseminated encephalomyelitis (n=52) Type 1 diabetes mellitus (n=6,191)Inflammatory bowel disease(n=3,388) Rheumatoid arthritis (n=4,685)	Whether chronic diseases in children were associated with their parents' employment, health care utilization, mental health, and mortality	Parents who have a child with a chronic illness tend to use health services more and also report higher levels of anxiety and depression. Furthermore, parents of children with chronic illnesses under the age of 50 without other complications have a 69% higher mortality risk. These parents are also more likely to require antidepressants or seek therapy with psychologists or psychiatrists. Regarding financial benefits, no differences were	Parental employment, mental health, and increased healthcare use were associated with chronic pediatric conditions. Pediatricians and primary care providers should consider the physical and mental health of parents with a child with a chronic condition.

				found among parents, but those who lived with a partner had a 25% higher risk of early retirement, which was not the case among single-parent families.	
Mark A. Ferro et al, 2021 [44]	Canada	Chronic physical illness	Described changes in psychological distress prior to and during the COVID-19 pandemic in parents and their children with chronic physical illness, compared parent-proxy and youth- reported perceptions of psychological distress and perceptions of COVID-19-related psychosocial health, and modeled factors predictive of psychological distress and psychosocial health among parents and youth.	Depressive symptoms, 9,8<br><br>CESD Parental stress, PSS 36,7<br><br>Family functioning, FAD 28,1<br><br>K6→preCoVID 4.0 Post 6.7<br><br>Illness severity, WHODAS 2.0→7,2	A substantial increase in symptoms of psychological distress has been observed from before to during the COVID-19 pandemic among young people with chronic physical illnesses and their parents.<br><br>Parental/family factors and online or blended learning environments were key risk factors in predicting worse outcomes in this vulnerable population of young people and their families. Family-centered models of care are needed to support the mental and psychosocial health of young people with physical illnesses and their families.

Ryo Morishima et al, 2021[83]	Japan	22q11.2 deletion syndrome<br><br>**Co-morbilitys**<br>Congenital heart disease<br>Immune system disorder<br>Endocrine disorder<br>Gastrointestinal disease<br>Otorhinolaryngology/ maxillofacial disease<br>Orthopedic disease<br>Growth/development al disorder<br>Psychiatric/neurologi cal disorder<br>Other	This study investigated relationships between medical, welfare, and educational challenges and paren- tal psychological distress.	Kessler 6→5.0	The study shows that the more comorbidities a child has, the greater the parental psychological distress. Furthermore, parents experience more stress when faced with difficulties accessing different medical services, when there is a lack of coordination and communication between welfare staff, problems managing school attendance, and disagreements between services and families in educational settings. Therefore, to provide better care to families with a child with 22q11DS, it is important not only to assess the severity of the child's medical conditions but also to understand the challenges parents face in medical, emotional, and educational aspects, in order to better support their mental health.
Inmaculada Riquelme et al, 2021 [57]	Spain	Cerebral palsy	To compare parental reports on family impact and healthcare satisfaction in children with CP with and without chronic pain and with and without speech ability	Parents reported moderate impact (mean = 69.14 (17.03), range = 26.39–96.53) and health- care satisfaction (mean = 64.96 (21.94), range = 2–100).	Pain affected the family's physical and social functioning, and the number of pain locations influenced parental concern. Families of children with chronic pain, both with and without verbal ability, reported the same impact on physical and social health. Greater pain intensity in the children produced a lesser impact on family communication. This suggests that the presence of pain may help promote pragmatic communication among family members to resolve emerging problems. Pain, and to a lesser extent, the ability to speak in children with cerebral palsy, can impact the physical, social, and psychological health of their

					families. Pain and, to a lesser extent, the ability to speak in children with cerebral palsy, can impact the physical, social, and psychological health of their families, although it does not appear to affect satisfaction with health care. Periodic assessment, health intervention, and family needs should be considered when designing family-centered programs for children with cerebral palsy.
A. Strzelczyk et al, 2021 [37]	Germany	Dravet Syndrome	To identify and describe factors that may have an impact on the disease burden of sufferers with DS and their caregivers	EuroQol 5D-5L 0,47	The disease is associated with a significantly reduced quality of life due to the following factors: seizure frequency, the high number of comorbidities, and therapeutic effort. All age groups had low rates. The adult rate, 0.47 (SD 0.17), was 0.41 lower than the general population rate, 0.88. The diagnosis of a child with Dravet syndrome has a significant impact on parents and siblings. One factor that helps cope with the socioeconomic and disease-related burdens is close family support.
Kelvin Ying et al, 2021 [48]	Malaysia	Cerebral Palsy	To examine the overall impact of caregiving for children with CP on the primary caregivers' health- related quality of life (HRQOL) and family functioning, and to identify potential factors	Parent HRQOL Summary Score 82,4 Family Functioning Summary Score 85,3 Total Impact Score 81,9. Through multiple linear regression analyses, the mother's level of education, family monthly income, sleeping problems in children with CP, and the existence of children with other types of disability have been identified as factors contributing to HRQOL and family functioning. The 3 highest subscale	Primary caregivers report a good quality of life and family well-being. Family functioning has a direct influence on the overall well-being of family members and the proper development of children with cerebral palsy.

			associated with primary caregivers' HRQOL and family functioning.	scores were the mean score for "family relationship" (M = 90.9, SD = 13.81), "social functioning" (M = 84.6, SD = 17.72) and "cognitive functioning" (M = 85.0, SD = 16.55). The lowest score was the mean score for "daily activities" (M = 76.1, SD = 23.84).	
Christine Settoon et al, 2021[84]	US	Children with tracheostomies	To measure the health literacy/numeracy rates in the caregivers of our pediatric tra- cheostomy population and the impact of the tracheostomy care on their quality of life	The average PTHSI score was 36 (SD 8.6), denoting a moderate level of caregiver burden. Nearly two-thirds of caregivers ranked their com- fort level for caring for their child upon hospital discharge as "very comfortable".	Low- and high-income caregivers showed no significant differences in caregiver burden, as measured by the PTHSI score. Health literacy rates among the general population are lower than health literacy results for caregivers of children with complex medical conditions. Assumptions about health, wealth, and caregiver burden are inaccurate, necessitating further evaluation.
Aycan Ünalp et al, 2022 [85]	Turkey	Epilepsy	To compare sociodemographic characteristics, quality of life, and levels of depression and anxiety of children with epilepsy and their families with a healthy control	**Beck Depression Inventory** Epilepsy group11,5 Control 9.<br>**Beck Anxiety Scale** Epilepsy group 6,5 Control 6.<br><br>**Short-Form 36 physical** Epilepsy 78,23 Control 86,47 **Emotional difficulty** Epilepsy 66,7 Control 100 **Mental** Epilepsy 65,13 Control 71,84 **General health** Epilepsy 62,83 Control 70,88.	Parents in the epilepsy group scored lower on quality of life questionnaires for physical health, emotional difficulties, psychiatric health, and general health than parents in the healthy control group.<br>There were no differences between parents in the epilepsy group and the control group on scales assessing parental depression and anxiety.<br>Families of children with chronic illnesses reported a lower quality of life than their children, and

			group.	(Statiscally significant)<br><br>Physical difficulty 75/100 Pain 76,02/77,30 Viability 65,96/65,68 Social function 75,875/77,00	families of healthy children reported better psychiatric health and well-being than their children.
Anna Rozensztrauch et al, 2022 [14]	Poland	Epilepsy	To assess the quality of life (QoL) in children with diagnosed epilepsy and the impact of a child's disease on the functioning of the family.	Parent QoL summary score 44,05 Family functioning summary score 46,94 ………………….. Total impact score 41,88	Children's medical conditions have a significant impact on family functioning. Parents included in the study reported the highest scores in the areas of cognitive functioning and family relationships, and the lowest scores in the areas of worry, daily activities, and emotional functioning.<br>Being a single parent has negative effects on physical and social functioning, as well as on their overall quality of life.
Merete K. Tschamper et al, 2022 [86]	Norway	Epilepsy	To investigate characteristics (sociodemographic variables, clinical variables of the child, parental self-reported self-efficacy, and level of perceived mental distress) associated with high and low levels of health literacy in	Self-efficacy 3.15<br><br>HSCL-10<br><br>Experienced mental distress 1,89	The only variable that predicted higher scores on the literacy scales was self-efficacy. Parental self-efficacy has been associated with greater parental responsiveness and sensitivity toward children and lower parental perceptions of distress.<br>Sick leave due to the child's epilepsy in the previous three months, comorbidities (in the child), and high levels of mental distress were associated with lower scores on several domains.

			parents of children with epilepsy.		
Karlie O'Brien et al, 2022[56]	US	Children undergoing gastrostomy tube placement	To identify changes in health-related quality of life over a 12-month period in the caregivers of these patients.	Physical functioning Hernia 83.2 G-tube 45,8<br>Emotional functioning Hernia 84.3 G-tube 47,8<br>Social functioning Hernia 89.0 G-tube 50<br>Cognitive functioning Hernia 80.8 G-tube 58,4<br>Communication Hernia 92.2 G-tube 46,3<br>Worry Hernia 86.3 G-tube 33<br>Daily activities Hernia 81.9 G-tube 20,3<br>Family relationships Hernia 92.0 G-tube 67<br>**Total HRQoL Hernia 86.0 Gtube 47,4**	Caregivers of children with gastrostomy tubes have a lower health-related quality of life (HRQoL) compared to caregivers of healthy children. Although their HRQoL improves over time, after 12 months they still have a lower quality of life compared to the control group. This indicates that the emotional and physical burden on caregivers remains significant over time, and suggests that targeted improvement initiatives to support caregivers after catheter placement would be useful.
Maja Brandt et al, 2022 [87]	Germany	Spinal Muscular atrophy	To review qualitative and quantitative studies on caregiver burden, the psychosocial situation, and family needs of parents as informal caregivers of children and adolescents with SMA.	**Psychosocial and burden-related outcomes for parents were: (health-related) quality of life (n = 10), caregiver burden (n = 7), caregiver time, (indirect) costs for families, parental stress, anxiety or depression symptoms, social support, coping (n = 2) as well as family needs, and satisfaction (n = 1).**<br><br>The most reported outcome for parents was (healthrelated) **quality of life**, with 63% (10 of 16) of studies reporting on this measure. Reduced quality of life was found in parents (a) with mental (46–84%) or physical (78%) health symptoms of their own [31, 35], (b) with financial problems due to care tasks (78%) [31], (c) of children and adolescents with more severe SMA subtypes (I–II) [33, 35, 38–40], and (d) shortly after a positive screening result for SMA [41]. parents of children and adolescents with SMA were more likely to have lower quality of life than the general population [16] or parents of children and	Research on parents caring for children with pediatric SMA reveals that they face numerous psychosocial challenges. These caregivers often experience a reduced quality of life, moderate to high stress levels, and a strain on their physical and mental health, especially when their children have SMA types I and II. Furthermore, they report a variety of unmet needs, including a lack of information, support in decision-making, integration into medical care, financial assistance, and support services.

				adolescents with other neuromuscular disorders [45]<br><br>In included studies using **caregiver burden** as an outcome, on average, most parents reported mild to moderate burden [15–17, 29], or high levels of stress due to their caregiving situation [32, 35]. The most frequently reported negative effects of caregiving on parents were sleep disturbances, physical or mental health symptoms of their own, changes in personal plans, work adjustments, and financial problems<br><br>One study reported high **anxiety and depressive symptom**s in 53% of mothers of children and adolescents with SMA, indicating slightly higher symptoms compared to mothers of adult patients with SMA [32]. One study measuring anxiety levels in parents shortly before nusinersen administration via lumbar punction (LP) of affected children and adolescents showed mild anxiety symptoms which decreased over time after the introduction of a self-developed psychological intervention [34]. Additionally, one study using the subscale 'anxiety/ depression' of the EQ-5D-5L reported that 65% of caregivers in a large Canadian sample showed at least some degree of anxiety or depressive symptoms with higher rates for parents of type I–II patients (69–74%) compared to those of type III patients (46%)<br><br>**Qualitative** findings of included articles could be divided into two main categories of psychosocial effects on caregivers: (1) family needs, caregiver burden, and psychosocial impact on parents as well as (2) experiences in the course of treatment (first symptoms, receiving the diagnosis, experiences with treatment and decision making).	
Vuong Prieto et al, 2022 [33]	US	Children with medical complexity	We hypothesized that a) increased caregiver burden would be	**Caregiver burden 25**<br><br>**Caregiving satisfaction 51,3**	The data show a negative association between caregiver burden and mental health and satisfaction with caregiving, and a positive association between

			negatively related to caregiving satisfaction and HRQOL, and b) caregiver burden and caregiving satisfaction would be associated with caregivers' gender, age, socioeconomic status, race/ethnicity, marital status, duration of caregiving, and number of other children living in the family, with the child's age, gender, primary diagnosis, duration of disease, number of hospitalizations, and type of health care technology needed.	**PCS52→SF12**<br><br>**MCS 45,6→12**	satisfaction with caregiving and mental health. Despite the burden being found in the study, many reported high satisfaction with caregiving. This highlights the importance of nursing staff identifying caregivers at risk of burden, as this can affect their well-being and satisfaction with caregiving, and interventions can be provided to provide them with the support they need.
Cibele Longobardi Cutinhola Elorza et al, 2023 [88]	Brazil	Chronic kidney disease	To investigate the presence of anxiety and depression and analyze the	WHOQOLbref 69.4 ± 13.0 for stage 3, 60.6 ± 17.6 for stage 4, and 48.1 ± 15.1 for stage 5.<br><br>BAI Mild Case group12 (41.4%) Control goup 9 (17%)	The results showed no differences in quality of life between the case and control groups using the WHOQOL-bref instrument. However, as the disease progressed, the families' environmental conditions

			quality of life of children and adolescents with age between 8 and 18 years with CKD stages 3, 4, or 5, and their primary caregivers and in healthy children and their PC. In addition, the study compared the results between groups, time of diagnosis, and stage of disease.	BDI Mild Case group 8 (27.6%) Control group 10 (18.9%)	worsened, including the availability and quality of healthcare and social care, and leisure opportunities.<br><br>Most caregivers in the case group presented mild anxiety symptoms, while anxiety was minimal in the control group. There were also differences between the two groups regarding depressive symptoms. Furthermore, there was a tendency to associate psychological well-being with emotional and physical aspects, although these associations were not very strong.
Florencia Epifani et al, 2023 [89]	Spain	Phosphomannomutase deficiency (PMM2-CDG)	Our study explores the adaptive profile of 37 PMM2-CDG patients, examining its association with parental stress and medical characteristics.	**Parental Stress Index**<br><br>Defensive response 60% Significant scores<br><br>Parental distress Children 52,4% Significant scores<br><br>Parent–child dysfunctional interaction Children 47,6% significant scores<br><br>Difficult child 33,3% significant scores<br><br>Total stress scale 47,6% significant scores	It was found that approximately 50% of caregivers experience clinically significant levels of stress, both in terms of feelings of parental distress, such as anxiety, depression, and burnout, and in relationship problems with their child, such as difficulties with discipline and communication. Furthermore, when the child has a more severe cerebellar motor syndrome, along with behavioral disturbances and other conditions such as hyperactivity, autistic traits, or moderate to severe intellectual disability, parental stress tends to be even greater.
Margarita Maltseva et al,	Germany	Dravet syndrome	To quantify sleep issues among	**PSQI** = 6 →76.9% (n = 83)→abnormal	Caregivers of individuals with DS show higher levels of anxiety and depression compared to the

2023 [90]		caregivers of patients with DS using the well-established Pittsburgh Sleep Quality Index (PSQI), which will allow us to contextualize and evaluate the severity of previously identified sleep and mental health issues experienced by caregivers.	sleep quality.<br><br>30.6% (n = 33) of caregivers, a score of 11 or above was achieved→severe sleep disorders.<br><br>The overall mean PSQI score of caregivers was 8.7 (SD 3.5; median 8.5; range 2–16), compared with a mean score of 5 (SD 3.4) among the general German population.<br><br>**HADS anxiety** normal (0–7) →38.2% (n = 39), borderline (8–10) →26.5% (n = 27), and abnormal 35.3% (n = 36), severe anxiety symptoms.<br><br>The overall mean HADS anxiety score was 9.3 (SD 4.3; median 9; range 1–20), compared with mean HADS anxiety scores of 4.4 (SD 3.3 for men) and 5.02 (SD 3.6 for women) among the general German population.<br><br>**HADS depression** normal (0–7) →49.1% (n = 53), borderline (8–10) →27.8% (n = 30), and abnormal→23.1% (n = 25), severe depression symptoms. The overall mean HADS depression score was 7.9 (SD 3.7; median 7; range 1–20), compared with mean HADS depression scores of 4.8 (SD 4.0 for men) and 4.7 (SD 3.9 for women) among the general German population.<br><br>**BSFC** score was 41.7 (SD 11.7; median	general German population, with 61.8% and 50.9% reporting elevated scores in these areas. Furthermore, caregivers who experience sleep problems or share a room with patients with DS tend to have higher anxiety levels. It was also found that more nocturnal seizures, transient seizures, and moderate sleep disturbances were related to higher stress and sleep problems scores. Sleep quality, anxiety level, and the severity of the patient's syndrome influence caregiver well-being.

				40) and ranged from 17 to 64, indicating a moderate overall care burden. The BSFC indicated a mild burden (0–41) for 54.6% of caregivers (n = 59), a moderate burden (4255) for 33.3% of caregivers (n = 36), and a severe or very severe burden (56–84) for 12.0% (n = 13) of caregivers.	
Hanna Skrobanski et al, 2023 [51]	UK	Tuberous sclerosis complex	To understand the wider household involvement in TSC care, and the non-healthcare-related costs and impacts on productivity for caregivers and their families.	**PedsQL FIM** Total Scale score = 43.3 (19.1); Family Functioning Summary score = 46.2 (23.0); and Parent HRQL Summary score = 45.4 (20.3). These scores were lower than those for a community sample of parents of children aged 2–17 years , caregivers of children with other conditions associated with developmental delay (e.g. cerebral palsy and attention deficit hyperactivity disorder) , and parents of children/adolescents with chronic pain. For the primary caregiver, the mean (SD) and median (range)<br><br>**HADS Anxiety and Depression** summary scores were 11.2 (4.8) and 10.5 (2.0–21.0) for anxiety and 7.9 (4.4) and 7.0 (0.0–19.0) for depression. Overall, 50% of primary caregivers were classified as having ‘probable clinical caseness’ for anxiety and 27% for depression. The summary scores for both measures were higher than the median UK population norms of 6 (women) and 5 (men) for anxiety and 3 for depression (both sexes)	The findings indicate that when seizures in children and adolescents with TSC are more frequent, caregivers spend more time caring for their children, which also negatively affects family quality of life and caregiver health. Furthermore, greater intellectual disability and the presence of neuropsychiatric comorbidities are associated with more time spent on caregiving and lower family functioning and caregiver quality of life. Primary caregivers feel they must always be available and only find time off when the child is at school or in someone else's care. This has impacted their work productivity and careers, as many have had to give up or reduce their work hours to be able to care for their children. Furthermore, a lower quality of life in children and adolescents with TSC is associated with more symptoms of depression in their caregivers.

Jessica Teicher et al, 2023 [91]	Canada	Children with medical complexity	To augment existing literature by exploring parents' experience of caregiving for CMC	Four major categories were identified to describe affected domains of parental caregiver experience: personal(identity, physical health, mental health), family(marriage, siblings, family quality of life), social(time limitations, isolating lived experience), and financial(employment, medical costs, accessibility costs)	Caregiving impacts personal, familial, social, and financial life. Resilience is fostered through the support of others and maintaining a positive outlook.
Kadi A Alhumaidi et al, 2023 [92]	Saudi Arabia	Cerebral Palsy	To assess it by developing a survey that targets the caregivers of children with CP.	Family Interaction (Scoring: 6-30)→23,48<br><br>Parenting (Scoring 5-25)→18,17<br><br>Emotional Well-Being(Scoring 4-20)→14<br><br>**Physical/Material Well Being(Scoring 4-20)→14,65**<br><br>**Disability-Related Support(Scoring 3-15)→10,60**<br><br>**Total Family Quality of Life Score(Scoring 22-110)→80,90**	Caregivers of children with cerebral palsy in Tabuk are generally very satisfied with family interaction and enjoy their bonds, despite the challenges they face. They also find satisfaction in parenting and helping children gain independence. Furthermore, they highly value the support they receive to help children with disabilities achieve their goals and make friends, which is very positive. However, some expressed dissatisfaction in certain areas, reflecting the emotional and practical challenges they face. Overall, aspects such as family interactions, parenting, emotional and physical well-being, and external support are critical to their well-being.
You Wu et al , 2023 [50]	China	Epilepsy	(1) to explore the interrelationships and evaluate the predictors of these issues; (2) to appeal to all epilepsy providers	**Family Burden of disease scale→ 16**<br><br>Family economic burden→5 Family daily activities→3 Family entertainment activities→3 Family Relationship→3 Physical health of family members →0 Mental health of	Parents of children with epilepsy and disabilities (CWE) face a very high burden of illness, especially financially, and also suffer from anxiety and depression, which significantly reduces their quality of life. The severity of these problems is related to factors such as a history

			to pay more attention to the patient and their family holistically than simply treating the disease; (3) to promote comprehensive management of CWE and improve their long-term outcomes.	family members→1<br><br>**GAD-7→7,5**<br><br>Normal→1→33%. Mild→7→27% Moderate→12→18,1% Severe→19→21,9%<br><br>**PHQ-9→6**<br><br>Normal→1→43% Mild→8→24,7% Moderate→11→13,2% Moderate Severe→16→8% Severe→24→11,1%<br><br>**WHOQOL-BREF→53,7**<br><br>Physical→57,1 Psychological→54,2 Social relationships→58,3 Environment→53,1<br><br>**PedsQL→65,7**<br><br>Physical functioning→75 Emotional→65 Social→70 School→60	of birth asphyxia, frequent seizures, comorbidities, and difficulties in children attending school. Furthermore, the older the child is at diagnosis, the more severe the parents' feelings of anxiety. The emotional and family burden affects both parents and children, but participation in school and outdoor activities can improve their quality of life. Therefore, it is recommended that healthcare professionals provide psychosocial and financial support to families, and promote social inclusion and participation in activities, to better care for these children and their families in a holistic manner.
Anna Rozensztrauchet al, 2023 [34]	Poland	Menkes Disease	To assess the quality of life of children with MD syndrome and the impact of the disease on family functioning.	**Caregiver burden** 4.88<br><br>**Perceived social support** 66.94<br><br>**PedsQL FIM** Family functioning—parents 44,16. Physical functioning—parents 39.84. Emotional functioning—parents 45.00. Social	The study found that children with Menkes disease (MD) have a poor health-related quality of life (HRQoL), especially in physical functioning, although their emotional well-being is relatively better. MD primarily affects cognitive functioning and family relationships, making it difficult for parents to concentrate and remember

				functioning—parents 41.80. Cognitive functioning—parents 50.00. Communication 48.44. Worry 35.31. Family functioning summary score 44.27. Daily activities 32.29. Family relationships 56.25.<br><br>**PedsQL children** Total score n=16 →27.40. Control n=6530→80,40	things. Furthermore, parents showed greater concern about aspects related to the family impact. The presence of comorbidities in children worsens their emotional well-being, causing them to experience more negative emotions and have more difficulty sleeping. Parents of children with MD face a moderate burden as caregivers, especially those who lack time for hobbies, feel socially isolated, lack close support, or have no contact with other families in the same situation. However, they also report high perceived social support, which can help them cope better.
Bethlyn Vergo Houlihan et al, 2023 [93]	US	Children with medical complexity	To effectively and creatively address the most urgent needs that matter to CMC and families, systems-level changes need to embed “lived experience.”	Although some emergent themes confirmed the current state of evidence we chose to focus on the 2 family-driven themes most salient for care system transformation.<br><br>Under **theme 1 (What’s missing – human dignity**), family subthemes describe feeling alone in the care journey because of societal bias and lack of dignity as having the greatest impact on their child’s QoL and family’s WB. **Theme 2 (What families really need and recommend in care)** addresses family subthemes of caring for the whole child and family, articulating what works, and does not, in practice, as well as essential but too often lacking ele- ments of care design.	CMC families expressed the need for system leaders to recognize, absorb, and transform the dehumanizing shortcomings of care delivery systems. Another prominent theme in the focus groups was the implementation of family-centered care and system redesign to achieve quality of life and overall well-being.
